# Wireworm-Associated Microbial Communities and their Implications on Biological Control

**DOI:** 10.1007/s00248-025-02672-4

**Published:** 2025-12-22

**Authors:** Adrian Wolfgang, Nora Temme, Ralf Tilcher, Mario Schumann, Gabriele Berg

**Affiliations:** 1 Institute of Environmental Biotechnology, Graz University of Technology, Petersgasse 12, Graz, 8010 Austria; 2 KWS SAAT SE & Co. KGaA, Einbeck, Germany; 3 Leibniz Institute for Agricultural Engineering and Bioeconomy (ATB), Max-Eyth- Allee 100, 14469 Potsdam, Germany; 4 Institute for Biochemistry and Biology, University of Potsdam, Karl-Liebknecht-Str. 24-25, 14476 Potsdam, Germany

**Keywords:** Metarhizium, *Agriotes* spp., Insect endosymbionts, Entomopathogenic fungi, Cuticle microbiome, Insect immune priming

## Abstract

Wireworms (larvae of different click beetles, *Elateridae*) are significant soil-borne pest species that can cause severe crop losses. They are difficult to control, and biocontrol using entomopathogenic fungi (EPF) display variable field efficacy. To understand microbial interactions and improve biological control, we studied the interplay between insect and soil microbiota in four wireworm species (*Agriotes* spp.) at temporal and spatial scales. We found that microbiota associated with wireworms are species-specific and primarily soil-derived. Our results further indicate that ectosymbiotic bacterial community composition on wireworm cuticles is relatively stable over time in specimens not deceasing from spontaneous entomopathogen infection. Therefore, successful microbiome homeostasis on cuticles appears to be correlated with long-term survival of wireworms in soil. Interestingly, EPF were prevalent but low-abundant in all wireworm species as well as in soils. Therefore, we analyzed immune priming effects by low-abundant EPF in soil. Mortality was higher in naïve wireworms than in wireworms pre-exposed to EPFs, and molting frequency increased, indicating both developmental adaptations and immune priming as strategies for EPF avoidance in wireworms. This work disentangles the key components of wireworm microbiomes and highlights the importance of microbial interactions for biocontrol. Biocontrol of wireworms could be improved by considering their species-dependency in microbiome homeostasis as well as physiological and behavioral adaptations to soil-borne pathogens. The potential functional synergies between EPF and soil microbes need further exploration.

**Supplementary Information:**

The online version contains supplementary material available at 10.1007/s00248-025-02672-4.

## Introduction

One of the most pressing challenges in agriculture is the higher crop loss resulting from globally increasing insect pest populations [1]. Simultaneously, the use of commonly used synthetic insecticides is being progressively phased out due to concerns regarding human and ecosystem health [2]. Hence, the demand for low-risk alternatives to insecticides is high [2, 3]. Microbial biocontrol agents are one of the key drivers towards a more sustainable agriculture [4, 5]. However, the implementation of microbial insect control strategies into common practice is progressing slowly and often struggles with crop-, pest species-, weather-, or site-specific variations in control efficacy [6]. These variations are particularly apparent when it comes to biocontrol of soil-dwelling insect pests, as these species are evolutionarily adapted to thrive in habitats characterized by high microbial diversity and abundance. Wireworms are a notable example of such soil-borne insect pests, posing significant challenges to crops such as potatoes, sugar beets, and maize in temperate regions [3, 7, 8].

Wireworms are larvae of click beetles (*Elateridae*), with approximately 1% of the species being of importance as agricultural pests [8]. Attempts to control wireworms using entomopathogenic fungi (EPF) such as *Metarhizium* spp. can be effective [9], but require improvement regarding efficacy and agricultural application strategies [6]. Interestingly, EPF were frequently found in field populations of wireworms without causing disease symptoms [10], indicating that additional factors beyond the sheer presence of EPF contribute to infection success in wireworms. Reciprocal adaptations of insect host and EPF may determine the outcome of insect-pathogen interactions. For instance, sublethal exposure to pathogens in insects can induce subsequent tolerance towards otherwise lethal infections, a process called immune priming [11]. To better understand the conditions that are required for successful EPF infections in wireworms, we have to consider the interplay between soil- and wireworm-associated microbes. We hypothesize wireworms to have developed such adaptations, that may be mediated via associated microbiota and/or their immune system.

Wireworms often occur as mixed populations on a given field site [12, 13], and the efficacy of *Metarhizium* spp. control varies among wireworm species [14]. The underlying mechanisms remain unclear; however, the phenomenon has been hypothesized to be related to variations in wireworm immune responses or their associated microbes [10]. EPF usually attach as spores to the cuticle of their future host before forming appressoria, followed by subsequent penetration of the cuticle, and proliferation in the host body [15]. Consequently, the insect cuticle is the primary physical and chemical interface for pathogen-insect interactions. This interface can also be colonized by epicuticular microbes that may interfere with EPF spore attachment or growth via niche exclusion, production of antifungal compounds, or the metabolization of cuticle compounds that would elicit spore germination [16, 17]. On the other hand, soil microbes could also facilitate EPF infection by producing cuticle-weakening or -degrading enzymes [18]. Identifying such microbes would also open the possibility for more targeted microbiome-based soil management to increase EPF efficacy. Additionally, wireworm species may host distinct symbiotic communities within their tissues, which could impact EPF virulence in host-internal tissues. It remains to be determined whether microbial associations with wireworms (a) vary among different wireworm species, and (b) given such differences occur, if they are found only on external surfaces (ectosymbionts), such as the cuticle, or only in internal tissues (endosymbionts), or in both. We hypothesize wireworm-associated microbes to differ between wireworm species, which may explain why we observe species-dependent efficacy of EPF under field conditions.

Wireworms typically have a life cycle ranging from 2 to 5 years, with certain species developing over a period of up to 11 years. They usually molt six to eight times before pupating [3, 8, 13]. Further, there is evidence for differences in EPF biocontrol efficacy depending on wireworm age and size [8, 19]. Given that epicuticular microbes play a role in biocontrol efficacy, the ability of certain soil microbes to colonize the new cuticle after a molting event is of importance for insect-pathogen interactions during ontogeny. The question remains, how stable or volatile cuticle-associated microbial communities are, and to what extent microbial wireworm symbionts are affected by the surrounding soil microbiome. We hypothesize that surrounding soil to some extent determines the structure of wireworm-associated microbiota, but the homeostasis of microbial communities over time to be crucial in wireworms for their survival.

The objective of this study is to describe bacterial and fungal microbiomes of four different wireworm species, distinguishing between ecto- and endosymbiotic colonizers, and comparing them to their respective home soil. Due to their long life cycle, we further investigate the stability of ectosymbiotic microbiomes over time. The occurrence of indigenous EPF led to another experiment to understand the effects of low EPF exposure followed by high EPF exposure on survival and molting frequency in wireworms. The overall goal of this study is to increase our ecological understanding of microbial dynamics on insect larvae with an emphasis on EPF-insect interactions.

## Methods

For the sake of conciseness, methods are briefly described. For a detailed description of the methods, please refer to “Detailed Methods” in the Supplementary Material of this manuscript.

### Wireworm Collection and Experimental Design

**Fig. 1:**
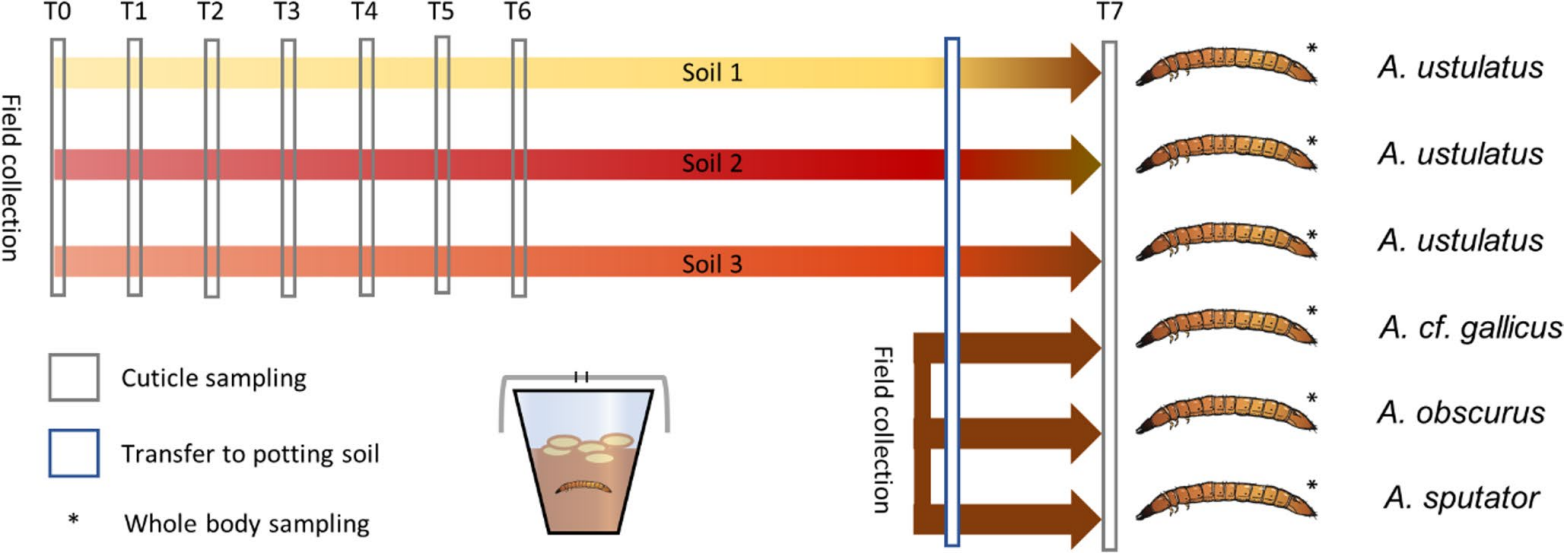
Schematic overview of the experimental setup and sample collection. Specimens were kept in microcosms, consisting of plastic cups with corresponding humid soil, covered with aluminum foil with a hole for air exchange, and supplemented with germinating wheat kernels as food. Ectosymbionts were sampled seven times (“cuticle sampling”) across several weeks. Before final ectosymbionts and endosymbionts DNA extraction (T7), all specimens were transferred to microcosms containing the same potting soil (see also Methods section)

Field collection 1 was conducted in April 2020 in Styria, Austria, using bait traps as suggested by [12] on three different fields; Field collection 2, using manual collection from field soil was performed weekly in Einbeck, Germany, from June to July 2020 to cover different wireworm species for the experiments (Supplementary Material: Methods Table M1). Wireworms were maintained separately (preventing cannibalism) in aluminum foil-covered plastic pots in their original soil. (Fig. 1) and fed with germinating wheat kernels.

For sampling ectosymbionts across time, soil loosely adhering to wireworms and gut content were removed using the method of [10], where wireworms are allowed to tunnel through saline agar overnight. Then, specimens were shaken by hand for 1 min in 1.2 ml washing solution (0.1% Tween 20, 0.9% NaCl, 30% glycerol). Soil from the field and wash suspensions were stored at -20 °C until DNA extraction. The process was repeated weekly for five weeks (T_0_-T_5_) with an additional sampling at day 92 (T_6_) for specimens from collection 1 (Fig. 1).

To investigate changes in ectosymbiotic microbiomes in different soil microbiomes, wireworms of collections 1 and 2 were transferred to microcosms (day 198) filled with potting soil substrate (Einheitserde ^®^ Classic Profisubstrat, Sinntal-Altengronau, Germany). Four 500 mg samples of substrate were directly taken for further amplicon analyses. After 19 days (day 217, T_7_), ectosymbionts of wireworms were sampled using a modified protocol after [20]. Then, wireworms were removed with a sterile tweezer, weighed, frozen in liquid nitrogen, and DNA extracted from the whole body using an adapted protocol of QIAamp Blood and Tissue Kit (Qiagen GmbH, Hilden, Germany).

### Wireworm Identification

Wireworms were identified morphologically based on traits described in [21, 22]. Three morphotypes were identified as *Agriotes obscurus*, *A. sputator*, and *A. ustulatus*. A random selection of 9 to 12 specimens per morphotype was made for subsequent analysis. To assess the ectosymbionts of the same specimens across time, three specimens per field of collection 1 (all *A. ustulatus*) were chosen. Wireworms were further identified using the method of Staudacher and colleagues [23]. Thirteen specimens could neither be identified based on this method nor by PCR amplification using standard COI primers LCO1490/HCO2198 [24], but with degenerated COI primers [25]. Wireworms were phenotypically and genotypically identified as *Agriotes* cf. *gallicus* (*n* = 9), *A. obscurus* (*n* = 3), *A. sputator* (*n* = 12), and *A. ustulatus* (*n* = 9). *A.* cf. *gallicus* specimens were initially morphologically identified as *A. obscurus*, as currently, there is no morphological description of *A. gallicus* larvae (personal communication Jörn Lehmhus [21]), ; Larvae of *A.* cf. *gallicus* could only be genotypically identified using the primers designed by [25].

### Isolation of Total-Community DNA and Illumina Sequencing

DNA of soil samples (*n* = 4/soil) was isolated using the FastDNA™ Spin Kit for Soil (MP Biomedicals, Heidelberg, Germany) following the manufacturer´s instructions. DNA of ectosymbionts and endosymbionts was extracted using an adapted and modified protocol from [20].

Amplicon PCR was conducted in 30 µl reactions using barcoded primer pairs 515f/806r [26] and ITS1f/ITS2r [27] for bacteria and fungi, respectively. PCR mixes included 2 x KAPA 3G Plant buffer and polymerase (KAPA Biosystems, Cape Town, South Africa), 0.3 µM of each primer, and 1 µl template DNA. For fungi, an additional 1.5µM MgCl_2_ was added. PCR cycling conditions were: 96 °C for 10 min, 35 cycles of 96 °C for 10 s, 54 °C (bacteria) or 58 °C (fungi) for 5 s, 72 °C for 15 s, and final elongation at 72 °C for 30 s. Extraction and PCR controls were always included. PCR products were sequenced by Novogene Co. Ltd. (Cambridge, UK) using Illumina NovaSeq 6000 250 bp paired-end reads sequencing.

### Real-Time Quantitative PCR of Wireworm-Associated Microbiomes

Real-time quantitative PCR was performed to assess microbial abundance in wireworms. It was conducted using three technical replicates in 10 µl reactions, containing KAPA SYBR^®^ Green 2X MM (KAPA Biosystems, Cape Town, South Africa), 5 pmol primers, and 1 µl template; in fungi, an additional 4 nmol MgCl_2_ was added. For ITS results, reads were measured as *Metarhizium robertsii* spore equivalents (MSE).

### Preprocessing and Preparation of the Dataset

Amplicon data were preprocessed in QIIME2 v.2022.02 [28]. Demultiplexing of raw amplicon sequences was performed using cutadapt [29]. Sequences were denoised using DADA2 [30], query databases for taxonomical assignment were SILVA v.132 [31] and UNITE v.7 [32] for bacteria and fungi, respectively. All analyses were performed in R v4.1.3 [33]. Chloroplast, mitochondrial, plant-assigned, and unassigned reads (domain/kingdom level) were removed. Mean alpha diversity metrics (species richness, evenness, Shannon) were calculated iteratively (*n* = 100) with random rarefying. For beta diversity comparisons, Bray-Curtis distances were calculated based on cumulative sum-scaled datasets.

### Amplicon Analyses

Alpha diversity indices were tested for normality using the Shapiro-Wilks test. Group comparisons of non-parametric data were performed using the Wilcoxon rank-sum test or Kruskal-Wallis test (FDR-corrected); for parametric data, we used the Student´s T-test and ANOVA (FDR-corrected). Beta diversity distance matrices were visualized as PCoA plots and analyzed using PERMANOVA [34] and pairwise PERMANOVA [35].

Biomarker taxa responding to a tested categorical variable were identified using linear discriminant analyses of effect size (LEfSe) [36]. *Metarhizium* spp. prevalence and relative abundance were calculated for each wireworm species separately in ectosymbiotic and endosymbiotic datasets.

To assess if microbial alpha diversity and abundance in wireworms is correlated with wireworm size, wireworm weight was used as an independent variable in linear regressions with alpha diversity metrics and qPCR results of ectosymbiotic and endosymbiotic microbiomes as dependent variables.

For tracking microbiome dynamics in ectosymbiotic microbiomes, the mean Shannon diversity of four *A. ustulatus* specimens residing in the same soil type was tracked across sampling time points T_0_-T_7_, where one specimen spontaneously deceased after five weeks. Bray-Curtis distances between soil samples and ectosymbionts were tracked over time points T_0_-T_7_ to evaluate if microbiome homeostasis is stable after molting.

To evaluate to what extent soil microbiomes determine wireworm ectosymbiont communities, ectosymbiont samples of *A. ustulatus* from timepoints T_0_-T_6_ were compared to timepoint T_7_, modeling alpha and beta diversity indices as a function of the origin soil. The package ’sourcetracker2’ [37] was used to estimate the proportions of microbes originating from the origin or potting soil.

### EPF Immune Priming in Wireworms

Three species of *Metarhizium* were used to assess whether non-lethal exposure of a given *Metarhizium* strain (= “Pre-treatment”) could affect the virulence of subsequent *Metarhizium* spp. challenges (“Treatment”) across EPF species; in other words, if low abundances of *Metarhizium* spp. induce immune-priming effects in wireworms. Wireworms were either naïve or challenged with low concentrations of *M. robertsii* in field soil before (“immune-primed”; challenge experiment is not part of this study). We exposed wireworms (*A. lineatus*, *n* = 16/treatment, provided by KWS SAAT SE & Co. KGaA) to high concentrations of EPF spores (3 species, *M. robertsii* strain R, *M. brunneum* strain B, *M. anisopliae* strain A, control group: 0.1% Tween 80). The three tested strains of *Metarhizium* did not show strong reciprocal growth inhibition in vitro (data not shown). Wireworms were pulse-vortexed in a 10^7^/ml spore suspension and kept singly in pots in a randomized order, containing the same untreated field soil as has been used for the pretreatment to minimize soil microbiome-dependent effects (natural *Metarhizium* spp. background load: 82 CFUs/g soil) and wheat seedlings as food for four weeks. This design resulted in eight treatment groups (Supplementary Material: Methods Table M2), with wireworms being separated across the entire experiment, allowing each specimen to provide independent responses to the pathogen challenge. Binomial testing was performed for each EPF strain separately to test whether mortality and the number of found cuticles differ due to (a) immune priming (all three *Metarhizium* spp. combined), and (b) species of the “treatment” EPF strain.

## Results

### Wireworms Harbor Less Diverse Microbial Communities than Soil

An overview of the amplicon dataset is given in Supplementary Table S1. The four soils tested partially differed in bacterial alpha (species richness: soil 1 > soil 2 > soil 3 > potting soil) and beta diversity indices (range R^2^_bacteria_ = 0.226–0.475; R^2^_fungi_ = 0.483–0.704), as well as bacterial and fungal biomarker taxa (Supplementary Table S2). Soil displayed higher alpha diversity indices (richness, evenness, Shannon) than wireworm-associated samples. All three tested compartments (soil, ectosymbionts, endosymbionts) significantly differed in bacterial (R^2^ = 0.23; *p* < 0.001) and fungal (R^2^ = 0.13; *p* < 0.001) community composition, with ecto- and endosymbiotic communities being more similar to each other than to the soil (Supplementary Table S3, Fig. S1). Quantitative real-time PCR analysis revealed a greater abundance of endosymbiotic bacteria and fungi compared to ectosymbiotic counterparts (Supplementary Table S4).

**Fig. 2:**
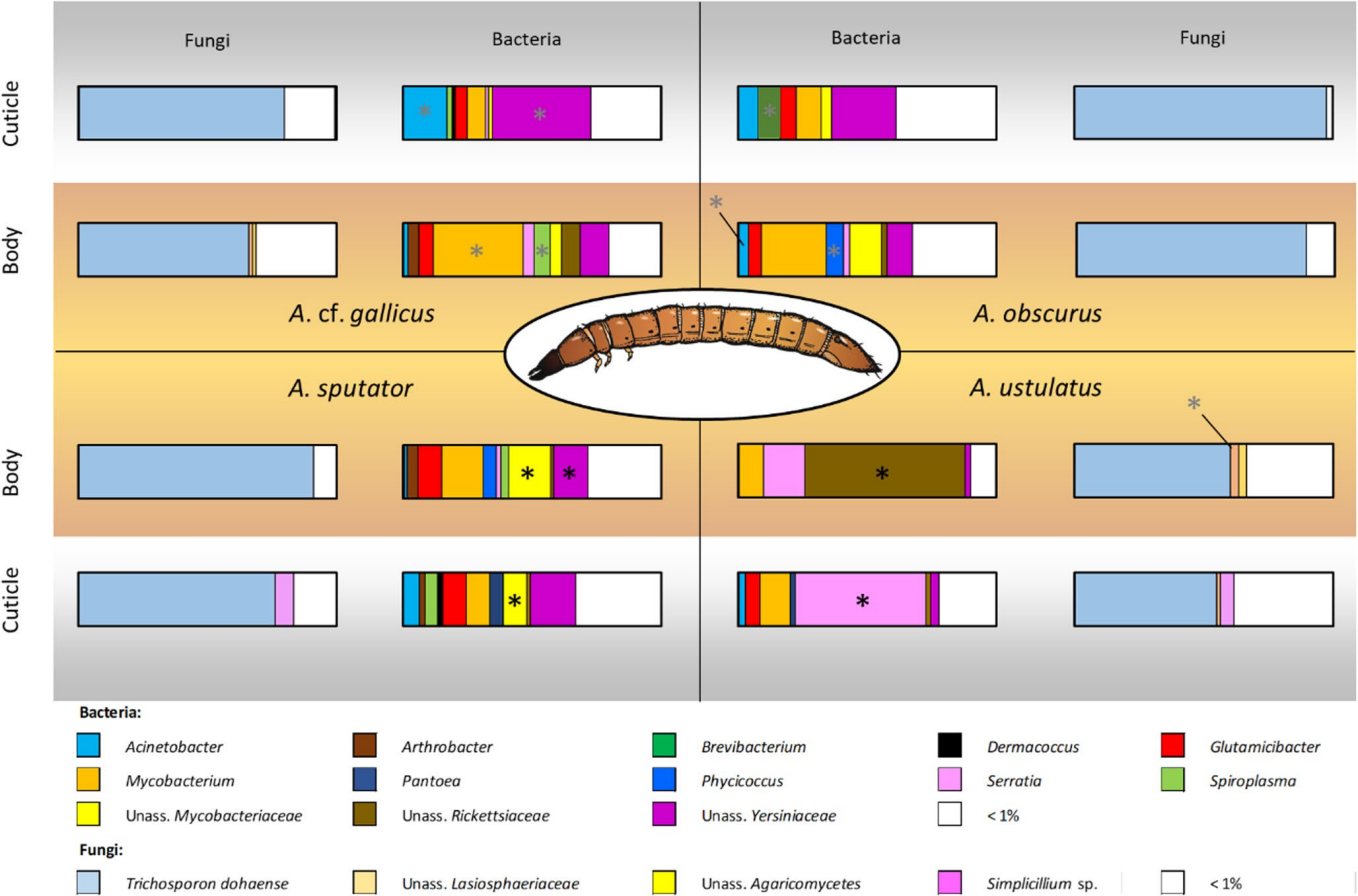
Microbial ectosymbiont and endosymbiont community composition of wireworms (*Agriotes* spp.). Stacked barplots displaying bacterial (vertically inner barplots) and fungal (vertically outer barplots) communities of ectosymbionts (grey background) and endosymbionts (orange background) of four different tested wireworm species (a). Asterisks in barplots: Potential biomarker taxa according to linear discriminant analyses of effect size (LefSe) for species-dependent differences in the respective compartment. Grey asterisks: biomarkers with *p*-value < 0.05 only if uncorrected for α errors. Sample sizes: n_*A*.cf.*gallicus*_ = 9; n_*A.obscurus*_ = 3; n_*A.sputator*_ = 12; n_*A.ustulatus*_ = 9

The wireworm microbiome contained numerous ASVs (bacteria: 49–859, fungi: 1-669), but was dominated by a few taxa, including *Yersiniaceae*, *Mycobacteraceae*, and *Trichosporon dohaense* (Fig. 2). 46–94% of all ASVs were unique to either ecto- or endosymbiotic communities; however, the sum of unique ASVs accounted for < 7% total relative abundance, except for fungi in *A. ustulatus* (ectosymbionts: 22%; endosymbionts 15%) and endosymbionts in *A. gallicus* (14.5%, Supplementary Table S5). ASVs assigned to *Metarhizium anisopliae* were prevalent in all tested wireworm species in ectosymbiotic (35 out of 58) and endosymbiotic (22 out of 35) samples, but in low (0.01–1.3%) relative abundances (Supplementary Table S6).

Ectosymbiotic communities were higher in bacterial species richness and Shannon diversity than endosymbiotic communities (Supplementary Fig. S1). Wireworm weight was negatively correlated with bacterial species richness, evenness, and Shannon diversity in both ectosymbionts (Supplementary Fig. S2) and endosymbionts (Supplementary Fig. S3), but not in fungi. Wireworm weight and microbial abundance were not significantly correlated (Supplementary Fig. S2, S3).

### Ecto- and Endosymbiotic Wireworm Microbiomes are Species-Dependent

**Fig. 3:**
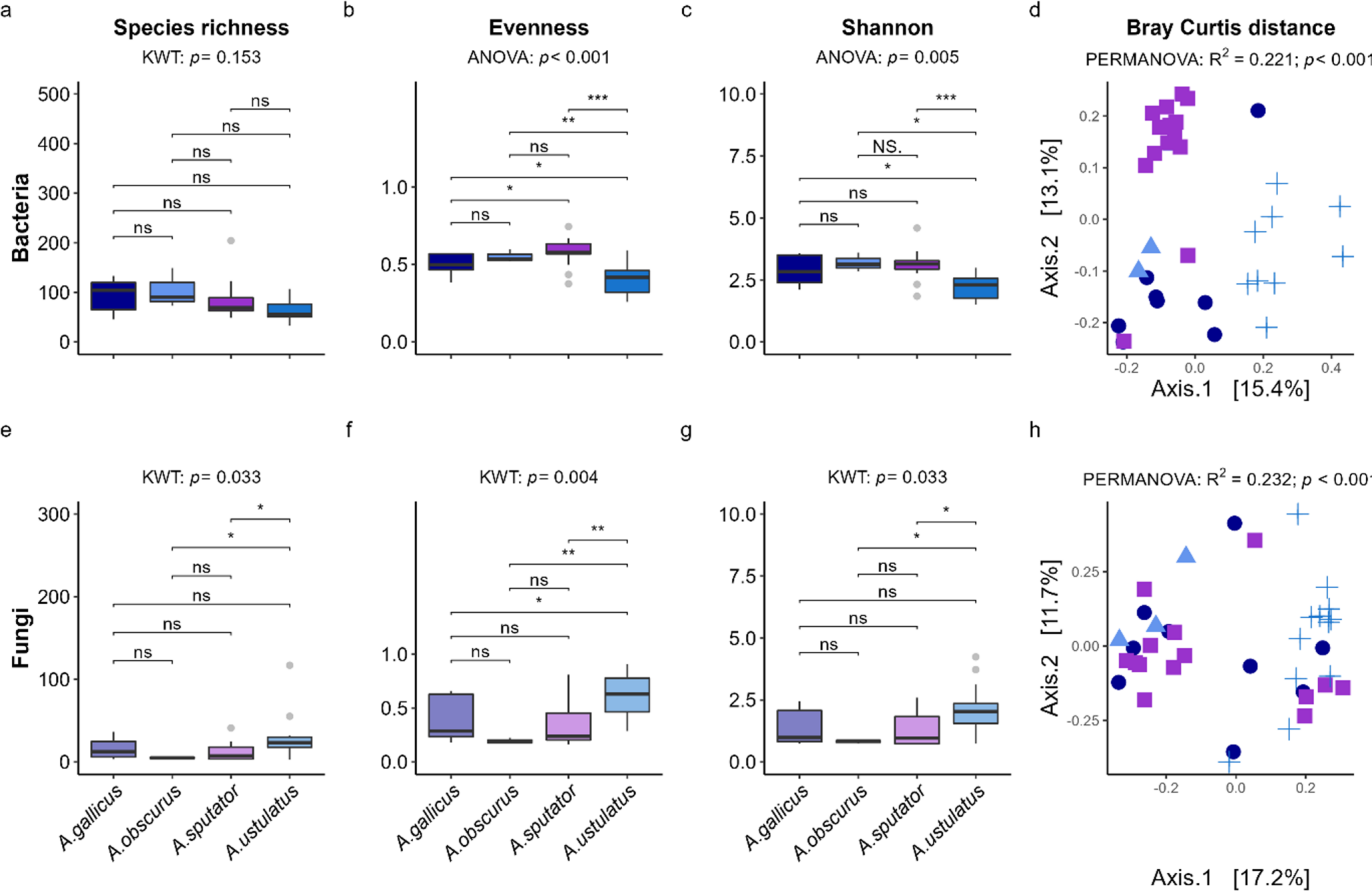
Overview of alpha and beta diversity in wireworm ectosymbionts. Species-dependent differences in bacterial (**a**-**d**) and fungal (**e**-**h**) species richness (**a**, **e**), Pielou‘s evenness (**b**, **f**), Shannon diversity (**c**, **g**), and community composition based on Bray-Curtis dissimilarity (**d**, **h**; PCoA plots). Sample sizes: n_*A*.cf.*gallicus*_ = 9; n_*A.obscurus*_ = 3; n_*A.sputator*_ = 12; n_*A.ustulatus*_ = 9; KWT: Kruskal Wallis test

We found wireworm species-dependent differences in wireworm microbiomes, both in ectosymbiotic and endosymbiotic microbiomes. Alpha diversity differences were mainly attributable to differences between *A. ustulatus* and all other tested wireworm species (Fig. 3, Supplementary Figure S4). Beta diversity significantly differed due to wireworm species in ecto- (Fig. 3d, h) and endosymbionts (Supplementary Fig. S4d, h). R^2^-values for the factor “species” were higher in ectosymbionts (R^2^_bacteria_ = 0.221, R^2^_fungi_ = 0.232) than in endosymbionts (R^2^_bacteria_ = 0.140, R^2^_fungi_ = 0.126). *A. sputator* and *A. *cf*. gallicus* originated from the same field site and were kept under the same conditions, yet displayed significant differences in community composition of ectosymbiotic bacteria (R^2^ = 0.14; *p* < 0.001; Fig. 3d), endosymbiotic bacteria (R^2^ = 0.12; *p* = 0.036), and endosymbiotic fungi (R^2^ = 0.07; *p* = 0.026). Biomarkers (LefSe, top five taxa, *p* adj. < 0.05) in ectosymbionts included *Variovorax* spp. for *A.* cf. *gallicus*; *Chryseobacterium* spp. and ‘Unassigned *Dermatophilaceae* spp.*’* for *A. obscurus*; ‘Unassigned *Mycobacteriaceae* spp.*’*,* Dermacoccus* spp., and *Knoellia* spp. for *A. sputator;* and *Serratia* spp. and *Smaragdicoccus* spp. for *A. ustulatus;* no ectosymbiotic fungal biomarkers were identified (Fig. 2, Supplementary Table S7). In endosymbionts, biomarkers included *Thermoplasmata* spp. for *A. obscurus*, ‘Unassigned *Mycobacteriaceae* spp.*’* and *Glutamicibacter* spp. for *A. sputator*, and ‘Unassigned *Rickettsiaceae* spp.*’* for *A. ustulatus* (Fig. 2, Supplementary Table S8).

### Ectosymbiotic Wireworm Microbiota Dynamics are Stable Under Healthy Conditions

**Fig. 4:**
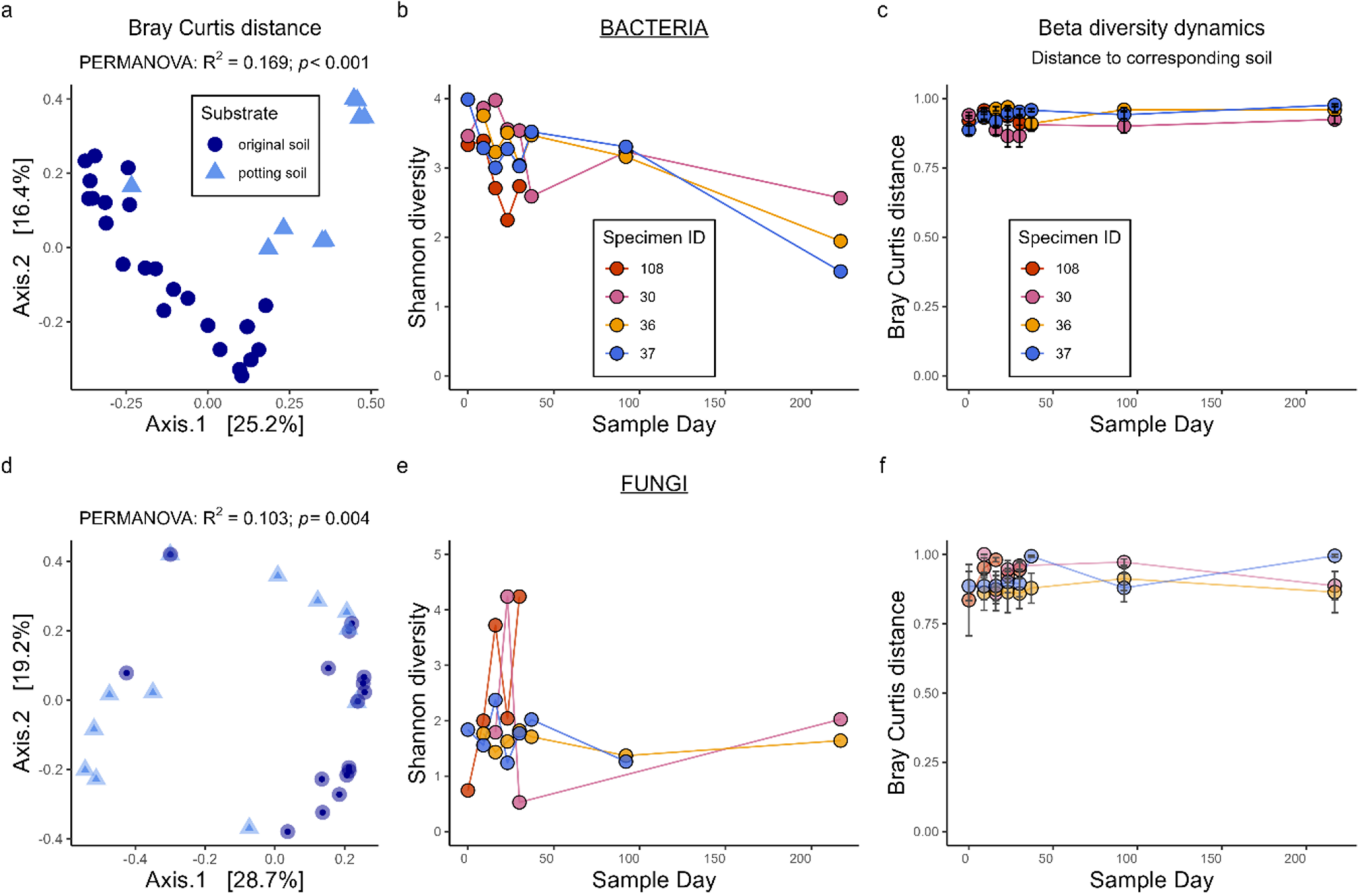
Dynamics in ectosymbiotic bacterial (top) and fungal (bottom) microbiomes. PCoA of Bray Curtis distances of ectosymbionts before (dark blue spheres) and after (light blue triangles) soil exchange (**a**, **d**); PERMANOVA results for Bray Curtis distances displayed on top. Volatility plot of Shannon diversity across time (**b**, **e**); Red spheres represent a specimen diseasing during the experiment due to spontaneous microbial infections. Bray-Curtis distances of ectosymbiotic communities to corresponding soil (mean value of four replicates) across time (**c**, **f**)

Ectosymbiotic community composition of *A. ustulatus* was compared before and after soil swap (see Methods: Wireworm Collection and Experimental Design; n_before_ = 25; n_after_ = 9), resulting in significant shifts in bacterial (R^2^ = 0.17; *p* < 0.001) and fungal (R^2^ = 0.10; *p* = 0.004) community composition if soil microbiome changes (Fig. 4a, d). Shannon diversity in bacterial and fungal communities before soil swap appears relatively stable over time for asymptomatic wireworms (here referred to as “healthy conditions”), except for the moribund specimen displaying a higher amplitude of bacterial and fungal Shannon diversity values (red spheres in Fig. 4b, e). Comparing Bray-Curtis distances of soil and ectosymbiont communities over time revealed that ectosymbiont communities consistently and strongly differ from soil communities (Fig. 4c, f).

### Legacy Effects of Soil Microbiota in Wireworms

**Fig. 5:**
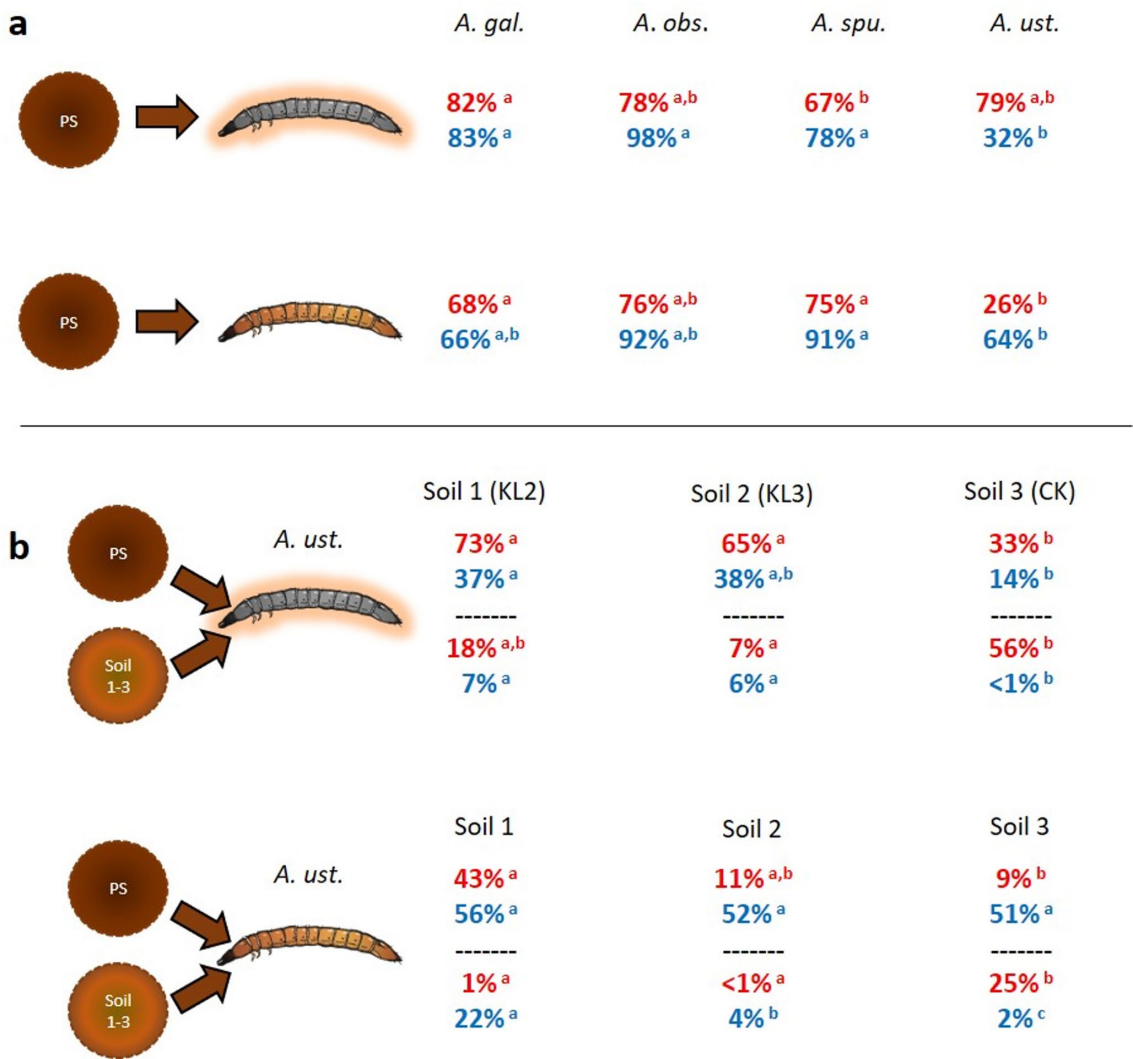
Wireworm microbes originating from soil. Proportions of potentially soil-derived bacteria (red) and fungi (blue) according to sourcetracker analyses. **a**: Estimated percentages of ectosymbiotic (top) and endosymbiotic (bottom) wireworm microbiota originating from substrate soil used in the laboratory; lowercase letters indicate differences between wireworm species based on the Wilcoxon rank sum test. **b**: For *A. ustulatus*, analyses were repeated using initial field soil (Soil 1, 2, or 3) and potting soil (PS) as possible sources of ectosymbionts (top) and endosymbionts (bottom). Lowercase letters indicate significant differences in the proportion of soil-derived microbes based on the pairwise Wilcoxon rank-sum test of sourcetracking results*.* *A. gal*.: *Agriotes* cf. *gallicus; A. obs.: Agriotes obscurus; A. spu.: Agriotes sputator; A. ust.: Agriotes ustulatus*

We compared ectosymbiotic and endosymbiotic bacteria and fungi between wireworms originating from different fields and subsequently being transferred to microcosms that all contained the same potting soil. “Original soil” only affected species richness and community composition of ectosymbiotic bacteria (Supplementary Table S9). Most ectosymbiotic and endosymbiotic microbes directly originate from the present soil in all wireworm species, except for ectosymbiotic fungi in *A. ustulatus* (Fig. 5a). In *A. ustulatus*, we could observe legacy effects of previous soil microbiomes (e.g., bacterial ectosymbionts from soil 3). Up to 56% (in soil 3 ectosymbionts) of wireworm-associated bacteria, and up to 22% of fungi (in soil 1 endosymbionts) originated from “original soil” microbiomes (Fig. 5b).

###  Pre-Exposure to EPF Affects the Mortality and Molting Behavior of Wireworms after Exposure to High Spore Concentrations 

Wireworms of the naïve control group did not show any mycoses. Immune priming without subsequent EPF spore challenge resulted in 3 mycosed out of 12 valid specimens (Supplementary Table S10). Pre-treatment with *M. robertsii* resulted in decreased mortality after exposure to high concentrations of *M. anisopliae* (Table 1); it did not significantly affect mortality for *M. brunneum* and *M. robertsii* treatments.

Molting frequency was significantly affected in all treatments. Pre-treatment with *M. robertsii* results in higher molting frequency in treatments with *M. anispoliae* and *M. brunneum*, but significantly lower molting frequency if wireworms were exposed to high concentrations of the same strain (Table 1).

**Table 1:** Binomial test results for the effect of immune priming on mortality and molting frequency of wireworms (*Agriotes lineatus*). Immune priming via low soil titre of *Metarhizium robertsii* in field soil (“primed”), subsequent exposure to high EPF spore concentrations (10^5^/ml suspension) of one of three tested *Metarhizium* spp. M.a.: *Metarhizium anisoplia*e; M.b.: *Metarhizium brunneum*; M.r.: *Metarhizium robertsii*. n: number of valid specimens per test group (pupae, imagos, and specimens with bacterioses removed from analyses); *p*: *p*-value of respective binomial test *: number adjusted, since more cuticles (*n* = 9) than specimens (*n* = 8) were found in the naïve group

Test (binomial)			*p*	Result
Mortality				
naïve + EPF (*n* = 30)	>	primed + EPF (*n* = 37)	**0.041**	Immune priming decreases mortality
naïve + M.a. (*n* = 11)	>	primed + M.a. (*n* = 13)	**0.047**	Immune priming decreases mortality
naïve + M.b. (*n* = 8)	>	primed + M.b. (*n* = 12)	0.139	no significant immune priming effect
naïve + M.r. (*n* = 11)	>	primed + M.r. (*n* = 12)	0.545	no significant immune priming effect
Cuticle				
naïve + EPF (*n* = 30)	>	primed + EPF (*n* = 37)	**0.018**	naïve wireworms molt more often
naïve + M.a. (*n* = 11)	>	primed + M.a. (*n* = 13)	**0.002**	naïve wireworms molt more often
naïve + M.b. (*n* = 8)	>	primed + M.b. (*n* = 12)	0.003*	naïve wireworms molt more often
naïve + M.r. (*n* = 11)	<	primed + M.r. (*n* = 12)	**0.009**	Immune-primed wireworms molt more often

## Discussion

Closely related beetle species can harbor very different microbiomes [38]. We found microbiota associated with wireworms to be highly specific, relatively stable in composition across time, and majorly soil-derived. While the sample size for some wireworm species is too limited (e.g., in *A. obscurus*) to generalize our results for all wireworm species of agricultural importance, our dataset provides a more detailed picture of their microbiological ecological context. Wireworms spend up to eleven years in soil [3, 23], and our results indicate that the cuticle represents a species-specific selective force for wireworm-associated microbiota. Ectosymbionts in insects can be translocated outside the body and reacquired after molting [39], which is supported by our data. Here, bacterial communities were found to be species-specific even in morphologically indifferent specimens (*A.gallicus* and *A. obscurus*); these differences were more pronounced in ecto- than in endosymbiont communities. However, more data is needed to support these results in other species and bigger datasets.

The majority of host-defending symbionts in insects are located on host surfaces or superficial structures [40]. Some coleopterans directly influence ectosymbiotic microbes. For instance, *Tribolium castaneum* directly secretes antifungal benzoquinones, interfering with EPF infection [41]. On the other hand, cuticle chemistry may select for protective microbiota from the environment [42], providing indispensable defensive functions for the host [43]. However, both direct and indirect effects on ectosymbionts may explain the varying efficacy of EPF in mixed wireworm populations [14]. Therefore, we hypothesize successful homeostasis of ectosymbiotic microbial communities to be crucial for the long-term survival of wireworms in soil.

The overall bacterial abundance and diversity in wireworms appear to depend on the corresponding soil [44], a finding which is supported by our results. Some taxa dominant in our wireworm amplicon datasets have been isolated before, e.g., *Acinetobacter* spp., *Arthrobacter* spp., *Mycobacterium* spp., *Pantoea spp.*, and *Serratia* spp [10, 44, 45]. Interestingly, all wireworm species contained several taxa assigned to *Actinobacteriota.* This taxon was identified as a defensive symbiont in several insects and other invertebrate taxa [40]. Similarly, *Serratia* includes defensive insect symbionts in aphids [40] and *Monochamus alternatus* [46]. *Rickettsiaceae* were highly abundant in *A. ustulatus*: depending on the host-microbe system, *Rickettsiaceae* can be pathogenic or mediate tolerance towards xenobiotics and toxins in insects [47]. Some insect pathogens are also potent producers of antimycotics [40], potentially mediating tolerance towards other entomopathogens. Therefore, we hypothesize that the high abundance of *Rickettsiaceae* in *A. ustulatus* is attributable to our experimental setup that included frequent exposure to saprobiotic fungi growing on moldy wheat kernels. Currently, the correlation between *Rickettsiaceae* and biotic stress protection in wireworms remains speculative and would require separate experiments generating additional data. Nevertheless, entomopathogenic microbes appear to represent a recurring part of wireworm microbiomes [44, 45].

*Trichosporon dohaense* dominates fungal wireworm microbiomes. The predominance of *Trichosporon* spp. in both ectosymbiotic and endosymbiotic wireworm communities could derive from a host-mediated selection or niche exclusion. Species of *Trichosporon* were found in insect guts before [48, 49]. Defensive fungal symbiosis has been described in fungus-farming ants, termites, and bark beetles [40]. As for bacteria, protective fungal symbioses may enable soil-dwelling insects to complete their long life cycle while being exposed to soil-derived pathogens: we observed potential entomopathogens (*Simplicillium* spp., *Metarhizium* spp.) in wireworm microbiomes. However, whether *Trichosporon* spp. mediate protective traits for pathogen tolerance in wireworms or simply can maintain itself in conditions found in and on the wireworm body remains speculative at this point.

We found that bacterial alpha diversity and wireworm weight are negatively correlated, and microbial abundance is not correlated with wireworm weight. Possible explanations include higher body-to-surface ratios, the time elapsed since the last molting event, or changes in cuticle chemistry during ontogeny. Previous investigations positively correlated EPF mortality with wireworm biomass [8], but this could also derive from a higher spore attachment probability [50]. Still, there may also be a mechanism that protects wireworms during their early developmental stages. For example, in *Paederus* spp. beetles, a symbiont-derived compound that deters predators is more concentrated in eggs and first instars, the otherwise most vulnerable life stages [39]. Currently, it is unclear if and how wireworms control their associated microbes during their whole ontogeny. Therefore, the negative correlation between bacterial alpha diversity and mortality risk needs bigger sample sizes and further evaluation.

Mortality due to *Metarhizium* spp. infections in wireworms is correlated with *Metarhizium* spp. abundance in soil [10]. Our experiments on immune priming suggest that wireworms adopted at least two distinct strategies in response to challenges posed by entomopathogens: immune priming and molting frequency adjustment. Sublethal exposure of insects to pathogens followed by an increased immune response is referred to as immune priming, commonly mediated by an increase in hemocyte density and antimicrobial peptide production by the insect host [11]. Such processes would explain the lower mortality in pathogen-challenged surviving wireworms compared to the naïve wireworms. However, we observed a much clearer response for the number of exuviae in soil than for mortality. Some insects utilize their exuviae as a food source following molting [51]; to our knowledge, there is currently no evidence that wireworms exhibit exuviae consumption. Consequently, we interpret the number of exuviae as an indicator of the number of molting events, which was higher in wireworms exposed to EPF. The removal of cuticle-attached EPF spores via molting would be a reasonable adaptation to escape EPF infection. This may also account for the observed variation in molting frequencies among *A. lineatus* populations from different fields [52], possibly being determined by the varying levels of EPF infection risk associated with the specific soil. Even if wireworms would usually consume their exuviae, our data indicate that wireworms avoid the ingestion of this potentially infectious material. However, both molting and immune-related physiological responses result in an energy trade-off. In *M. robertsii* treatments, mortality did not differ, but molting frequency was higher after pre-treatments. We hypothesize that these specimens invested more energy in molting despite immunological preconditioning. *M. robertsii* was the most virulent tested strain (data not shown); consequently, increasing molting frequency may be a short-term emergency strategy and, in this case, a more effective defensive strategy than immune priming. In any case, the low-abundant prevalence of EPF in wireworms we observed in our amplicon dataset appears to have physiological consequences and thus implications for wireworm biocontrol.

## Conclusion

This study characterized the bacterial and fungal communities found in four wireworm species, revealing species-specific bacterial community compositions. These species-dependent differences were more pronounced in ectosymbionts than in and endosymbionts, indicating the importance of the cuticle as a physicochemical barrier towards soil-borne pathogens. We further identified new microbial target taxa for future microbiome modulation approaches, for in vitro antagonism tests, or for field experiments aimed at improving biocontrol efforts directed towards this pest species complex. In addition, our results indicate frequent natural exposure of wireworms to low abundances of entomopathogenic fungi. This appears to lead to immune priming and molting frequency adaptations in wireworms, thus affecting biocontrol efficacy when using entomopathogens. Microbial communities in wireworms appear to be greatly influenced by the soil microbiome. Therefore, indirectly modulating wireworm-associated microbiomes through soil microbiome management and designing functional synergies between EPF and soil microbes may lead to strategies for sustainable and more targeted wireworm control.

## Supplementary Information

Below is the link to the electronic supplementary material.

Supplementary Material 1

Supplementary Material 2

Supplementary Material 3

## Data Availability

The datasets supporting the conclusions of this article are available at the European Nucleotide Archive (ENA, https://www.ebi.ac.uk/) under the accession PRJEB58336.
