# Supplementary material for: Wireworm-Associated Microbial Communities and their Implications on Biological Control
Source: Microb Ecol. 2025 Dec 22;89:31. doi: 10.1007/s00248-025-02672-4 (PMC12808278; doi:10.1007/s00248-025-02672-4)
Supplement: Supplementary file 1 [file 248_2025_2672_MOESM3_ESM.pdf]

Supplementary Material

Wolfgang et al., (2025): "Wireworm-associated Microbial Communities and Their Implications on Biological Control".

AUTHORS: Adrian Wolfgang, Nora Temme, Ralf Tilcher, Mario Schumann, Gabriele Berg

<u>Content</u>

Detailed Methods:

- **Wireworm collection and maintenance**
- **Experimental design and sampling procedure**
- **Wireworm identification**
- **Isolation of total-community DNA and Illumina sequencing**
- **Real-time quantitative PCR of wireworm-associated microbiomes**
- **Preprocessing and preparation of amplicon dataset**
- **Amplicon analyses**
- **EPF immune priming in wireworms**
- **In vitro antagonism of different Metarhizium strains**
- **Additional References**

Supplementary Figures:

- **Figure S1**: Differences in bacterial and fungal diversity of soil, ecto- and endosymbionts
- **Figure S2**: Alpha diversity and microbial abundance as a function of wireworm size in ectosymbiotic communities
- **Figure S3**: Alpha diversity and microbial abundance as a function of wireworm size in endosymbiont communities.
- **Figure S4**: Overview of alpha and beta diversity in wireworm endosymbionts.

Supplementary Tables:

- **Table S1**: Overview of filtered amplicon dataset
- **Table S2**: Differences in soil diversity used in the current study
- **Table S3**: Pairwise comparisons of bacterial and fungal community composition between the tested compartments
- **Table S4**: Summary of RT-qPCR results for 16SrRNA and ITS reads in wireworms
- **Table S5:** ASVs shared between ectosymbiotic and endosymbiotic communities based on VENN analyses.
- **Table S6:** Prevalence and abundance of the genus Metarhizium in four field-collected wireworm species
- (**Table S7**: Top five potential biomarkers for wireworm species in wireworm ectosymbionts according to LefSe analyses ➔ see separate file
- **Table S8**: Top five potential biomarkers for wireworm species in wireworm endosymbionts according to LefSe analyses ➔ see separate file)
- **Table S9**: Potential legacy effect of the origin soil on *Agriotes ustulatus* microbiome diversity after soil swap
- **Table S10**: Test groups and raw data for the experiment regarding immune priming in wireworms

Detailed Methods

DETAILED: Wireworm collection and maintenance

Wireworm specimens were collected from four different, yet unplanted vegetable fields on two collection dates in April 2020 in Styria, Austria (**Table 1**), using traps as suggested by MELES GmbH (https://www.melesbio.at/drahtwurm-koederfalle/, St. Pölten, Austria). A total of 120 wireworms were collected from traps, further maintained singly to prevent cannibalism. Wireworms were transferred to 40 ml plastic cups filled with ca. 20 ml soil (corresponding to wireworm origin, sieved, macroscopic invertebrates removed) and five pre-soaked wheat kernels as food (16 ± 2°C, 60% RH). Cups were covered with aluminum foil with a centered 5mm hole to impede wireworm escape and soil dehydration, but allow gas exchange and wheat growth, and are further called "microcosms" (**Figure 1**). Microcosms were watered occasionally if the soil visually appeared dry, and moldy plant materials were replaced with new kernels.

A second wireworm collection was carried out weekly in Einbeck, Germany, from June to July 2020, to cover different wireworm species (**Methods Table M1**). Specimens were stored in peat soil at 4°C with potato slices as potential food and humidity sources until use.

Methods Table M1: Collection of wireworm specimens. Source, date, and coordinates of sampling sites. For soil 1-3, field soil was collected for rearing wireworms in their corresponding soil. *: Trap designed after MELES GmbH (https://www.melesbio.at/drahtwurm-koederfalle/, St. Pölten, Austria)

	**Coordinates**	**Crop of previous year**	**Crop**	**Sampling date**	**Catch method**	**# total Wireworms caught**	**# of traps**	**Identified species**
**Soil 1 (KL2)**	47°00'51", 15°28'04"	Carot	yet unplanted	20/04/2020	Wheat trap*	36	*5*	*A. ustulatus*
**Soil 2 (KL3)**	47°00'51", 15°28'07"	asparagus, black oat	asparagus	20/04/2020	Wheat trap*	44	*5*	*A. ustulatus*
**Soil 3 (CK)**	47°05'43", 15°47'08"	potato	winter wheat	21/04/2020	Wheat trap*	32	*15*	*A. ustulatus*
**Soil 4 (KWS)**	51°49'15", 9°51'10"	grassland	Sugar beet	June-July 2020	Direct collection from soil	91	*NA*	*A.* cf. *gallicus*
								A. obscurus
								A. sputator

DETAILED: Experimental design and sampling procedure

Following the first collection (day 0, T_0), wireworms were individually placed in Petri dishes with physiological saline agar (0.8% agar, 0.9% NaCl to prevent osmotic stress) and stored overnight. Loosely adhering soil particles and wireworm gut content are removed as specimens tunnel through the medium [1]. Then, specimens were washed in a 1.5ml Epi with 1.2ml washing solution (0.1% Tween 20, 0.9% NaCl, 30% glycerol) by shaking for 1min, and returned to their respective microcosm. The washing solution was stored at -20°C until further processing. Agar tunneling and subsequent washing steps were repeated weekly, five times (T_0-T_6). Each time point included a separate extraction control sample.

To investigate changes in ectosymbiotic microbiomes in different soil microbiomes, wireworms of both collection dates were transferred to newly prepared microcosms (day 198, T_7) filled with a 1:1:1 mixture of sterile sand, sterile vermiculite, and non-sterile potting soil (Einheitserde ® Classic Profisubstrat, Sinntal-Altengronau, Germany). Four 500mg samples of potting soil were directly taken for further amplicon analyses and stored at – 20°C until DNA extraction. After 19 days in the new soil (day 217, T_7), wireworms were vortexed in 1.5ml Eppendorf tubes with 300µl extraction buffer A (20mM Tris, 2mM EDTA disodium dihydrate, 1.2% Triton X-100, pH 8) for one minute. Then, wireworms were removed with a sterile tweezer, frozen in liquid nitrogen, and stored at -20°C until DNA extraction.

DETAILED: Wireworm identification

Wireworms were first identified morphologically based on traits described in [2, 3] and references therein (between T_6 and T_7). Morphological identification resulted in three different wireworm morphotypes, identified as *Agriotes obscurus* L., *A. sputator* L., and *A. ustulatus* Schaller. Nine to twelve specimens of each morphotype were randomly chosen for further processing; for specimens from the first collection (all identified as *A. ustulatus*), three specimens per soil type were selected. Wireworms were further identified using molecular methods; Following whole body extraction of DNA (next section), the multiplex-PCR technique developed by Staudacher and colleagues [4] was

utilized. Thirteen specimens could neither be identified based on this method nor by PCR amplification using standard COI primers (LCO1490/HCO2198 [5]), but with degenerated COI primers [6]. Amplifications were checked using gel electrophoresis, PCR products were purified using "Wizard SV Gel and PCR Clean-Up System" (Promega, Madison, WI, USA), Sanger sequenced by LGC Genomics (Berlin, Germany), and identified using the nr/nt database and the BLAST (https://blast.ncbi.nlm.nih.gov/) with standard settings.

DETAILED: Isolation of total-community DNA and Illumina sequencing

DNA of soil samples (n = 4/soil) was isolated using the FastDNA™ Spin Kit for Soil (MP Biomedicals, Heidelberg, Germany) according to the manufacturer's instructions with an elution volume of 200µl. DNA extraction of ectosymbiont and endosymbiont DNA was performed using an adapted and modified protocol from Birer *et al.* (2017). For longitudinal ectosymbiont samples (T_0-T_6), samples were centrifuged for 20 min. at 13,500 g, the pellet including 270µl wash solution was vortexed with 30µl of 10x extraction buffer A (200mM Tris, 20mM EDTA disodium dihydrate, pH 8). Ectosymbiont samples after soil swap (T_7) were directly extracted in 1x extraction buffer A. For extracting endosymbionts, frozen wireworms were weighed and homogenized in bagmixer bags with 400µl extraction buffer A using a mortar and pestle. 300µl of suspension was transferred to a sterile 2ml Eppendorf tube. Subsequent steps were performed for all wireworm-associated samples. Suspensions were incubated at 37°C for 1h with 7.5mg lysozyme from hen egg white (Sigma-Aldrich, Steinheim, Germany). Samples were further processed using QIAamp DNA Minikit (Qiagen GmbH, Hilden, Germany) following the manufacturer's instructions, starting by adding buffer AL and proteinase K. Elution volume of buffer AE was 50µl for ectosymbiotic, and 200µl for endosymbiotic samples. All DNA extraction contained control samples.

Amplicon PCR was conducted in 30µl reactions using barcoded primer pairs 515f/806r [8] targeting the 16SrRNA region, and ITS1f/ITS2r [9] targeting the ITS region of bacteria and fungi, respectively. PCR mixes included 2 x KAPA 3G Plant buffer and polymerase (KAPA Biosystems, Cape Town, South Africa), 0.3 µM of each primer, and 1 template DNA. If amplification was poor, template

volume was adjusted (≤5µl). For fungi, an additional 1.5µM $MgCl_2$ was added to increase PCR efficiency. PCR cycling conditions were: 96°C for 10 min, 35 cycles of 96°C for 10 s, 54°C (bacteria) or 58°C (fungi) for 5 s, 72°C for 15 s, and final elongation at 72°C for 30 s. PCR controls were included.

Amplification success was checked using gel electrophoresis, and PCR products were purified using "Wizard SV Gel and PCR Clean-Up System" (Promega, Madison, WI, USA). DNA concentrations were estimated with Nanodrop 2000 (Thermo Scientific, Wilmington, DE, USA), and PCR products were pooled in equimolar concentrations and sequenced by Novogene Co. Ltd. (Cambridge, UK) using Illumina NovaSeq 6000 250bp paired-end reads sequencing.

DETAILED: Real-time quantitative PCR of wireworm-associated microbiomes

Real-time quantitative PCR was performed with three technical replicates in 10µl reactions on a Rotor-Gene 6000 device (Corbett Research, Mortlake, Australia). The reaction mix contained KAPA SYBR® Green 2X MM (KAPA Biosystems, Cape Town, South Africa), 5 pmol primers, and 1µl template, in fungal PCR mixes, additionally 4 nmol $MgCl_2$. For bacteria, cycling conditions were 95 °C for 3 min, 40 cycles of 95 °C for 5 s, 54 °C for 20 s, and 72 °C for 5 s. For fungi, cycling conditions were 95 °C for 5 min, 40 cycles of 95 °C for 20 s, 58 °C for 15 s, and 72 °C for 30 s. In fungi, DNA extracted from a *Metarhizium robertsii* M33 spore suspension (institute-internal strain collection) with known spore number was used as a standard; results for ITS reads are therefore displayed as *Metarhizium* spore equivalents (MSE). Mean fragment copy numbers were blank-corrected. Since we observed mitochondrial, plastid DNA (16SrRNA dataset), unassigned, and plant-assigned reads (ITS dataset) in amplicon sample results, the corresponding relative abundance in the amplicon dataset was used to remove non-target reads from qPCR data.

DETAILED: Preprocessing and preparation of the dataset

Amplicon data were preprocessed in QIIME2 v. 2022.02 [10]. Demultiplexing of raw amplicon sequences was performed using cutadapt [11]. Sequences were denoised using DADA2 [12] and truncated at 160bp and 140bp for bacteria and fungi, respectively. Taxonomical assignment of amplicon sequence variants (ASVs) was conducted using VSEARCH [13] with SILVA v.132 [14] and UNITE v. 7 [15] databases as bacterial and fungal reference sequences, respectively. ASV table, taxonomy, and metadata were further processed using the 'phyloseq' package [16] in R v4.1.3 [17]. For the bacterial dataset, chloroplast, mitochondrial, and unassigned reads (domain level) were removed. For fungi, unassigned (kingdom level) and plant-assigned reads were removed. Bacterial and fungal contaminants were detected using both prevalence- and frequency-based methods implemented in 'decontam' [18] utilizing PCR and extraction control samples. Rarefaction curves (**Methods Figure M1**) were calculated using the 'ranacapa' package [19]. To estimate species richness, Pielou's evenness, and Shannon diversity, a cut-off for rarefying was set to 960 and 245 for bacteria and fungi, respectively (seven fungal samples were removed). The diversity indices were calculated 100 times with randomly rarefying, and the arithmetic mean of the calculated indices was used for further analyses. For beta diversity comparisons, Bray-Curtis distances were calculated based on cumulative sum-scaled datasets.

Methods Figure M1: Rarefaction curves of bacterial (a) and fungal (b) datasets. Potential subsampling thresholds are displayed as vertical lines. Choosing a threshold of 960, all bacterial samples could be retained. For fungi, the threshold was set to 245, with seven samples not meeting the requirements, but covering estimated species richness.

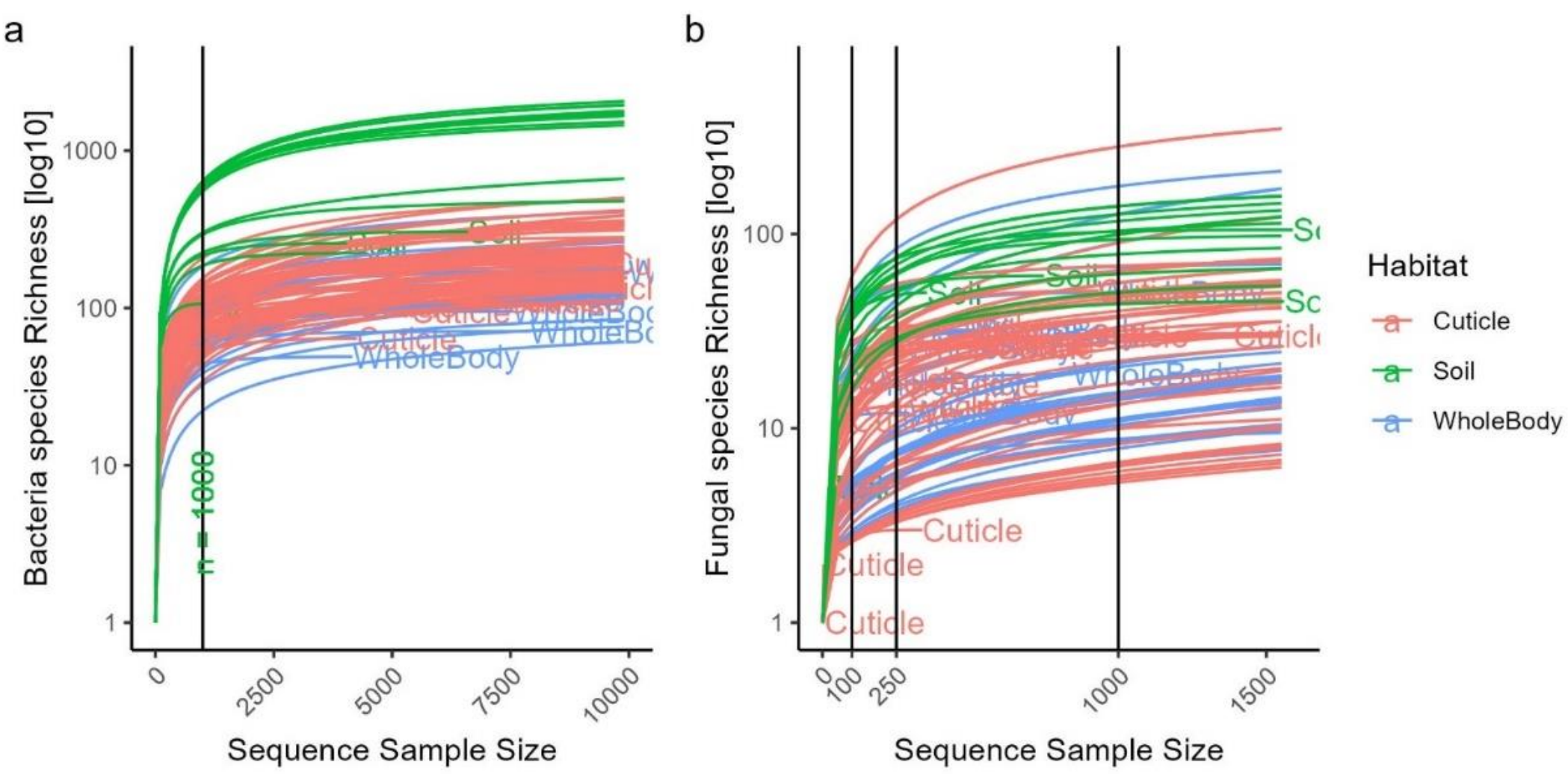

DETAILED: Amplicon analyses

Alpha diversity indices were tested for normality using the Shapiro-Wilks test. If not otherwise stated, analyses of parametric data were performed using the Wilcoxon rank-sum test (pairwise comparisons) or Kruskal-Wallis test (> two-group comparisons) with subsequent FDR correction; normal data were analyzed using the T-test (two-group comparisons) and ANOVA (> two-group comparisons) with subsequent FDR correction. Distance matrices for beta diversity analyses were visualized as PCoA plots and analyzed using PERMANOVA [20] and pairwise PERMANOVA [21]. To identify biomarker taxa responding to a tested categorical variable, linear discriminant analyses of effect size (LEfSe) [22, 23] were used.

To test for wireworm species-dependent differences, alpha (species richness, evenness, Shannon diversity) and beta diversity indices (Bray Curtis distances) were modeled with "wireworm species" as the dependent variable. Both ectosymbionts and endosymbionts were tested separately for biomarker taxa using LEfSe. To evaluate whether ectosymbiotic and endosymbiotic microbiomes contain different microbe species, ASVs found in ectosymbiont and endosymbiont microbiomes were analyzed separately using Venn (http://bioinformatics.psb.ugent.be/webtools/Venn/) for each wireworm species. To assess *Metarhizium* occurrence in different wireworm species, prevalence and relative abundance were calculated for ectosymbiotic and endosymbiotic wireworm microbiomes.

Wireworm weight was used as a proxy for size and used as an independent variable in linear regressions with species richness, evenness, Shannon diversity, and microbial abundance (qPCR results) of ectosymbiotic and endosymbiotic microbiomes as dependent variables.

For tracking microbiome dynamics in ectosymbiotic microbiomes, the mean Shannon diversity of four specimens residing in the same soil type was tracked across all sampling time points (T_0-T_7), with one specimen spontaneously deceasing after five weeks. Bray Curtis distances between soil samples (n = 4) and ectosymbionts were tracked over time to evaluate if community composition distances to soil microbiomes decreased or re-established after molting.

To evaluate if soil swap changes ectosymbiont communities, ectosymbiont samples of *A. ustulatus* from timepoints T_0-T_6 were compared to timepoint T_7, modeling alpha and beta diversity indices as a

function of origin soil. The package ’sourcetracker2’ [24] was used to estimate the proportions of microbes originating from origin or potting soil. Source and sink rarefaction depths were set to the minimum corresponding sample reads but with a maximum value of 1000.

DETAILED: EPF immune priming in wireworms

Field soil from sampling point KL1 was sieved (1cm); stones and plant debris were removed. Indigenous *Metarhizium* abundance in this soil was assessed using ten biological replicates and a dilution series with 0.1% Tween on EPF-selective SDA plates [25]. SDA plates were incubated at 19°C for 14 days; colonies with white hyphae and green-to olive-colored spores were regarded as *Metarhizium*-like CFUs (mrlCFUs). The assessment yielded a baseline abundance of 82 mrlCFUs/g soil. Shortly, different fodder plants for the wireworms were planted in soil from KL1 in a planting hole supplemented with 10^5 *Metarhizium robertsii* strain R spores in 0.1% Tween 80 (control group: only 1ml 0.1% Tween 80) and grown under greenhouse conditions for five weeks. Then, wireworms were added (1 per pot) and kept in this soil for another 2 weeks. Wireworms (*A. lineatus*), that in this way were indirectly exposed to a *Metarhizium robertsii* strain R (internal institute collection of Institute of Environmental Biotechnology, Graz, Austria) in this specific soil and survived the exposure (not part of this study), were defined as “immune-primed” group; specimen that were not exposed to this particular strain were defined as “naïve”. We expected wireworm-associated microbiota to have been affected by the new soil microbiome, but due to this standardized experimental setup we expect the wireworms to be similarly affected across treatment groups, except for the different EPF concentrations. Using this experimental setup, both control and treatment groups were kept under the same conditions, but with and without artificially increased EPF abundance. Naïve and immune-primed wireworms were pulse vortexed in a 2ml Epi containing 1 ml of a 10^7 spores/ml 0.1% Tween 80 suspension of three different *Metarhizium* strains: *M. robertsii* strain R, *M. brunneum* strain B, *M. anisopliae* strain A (all internal institute collection of Institute of Environmental Biotechnology, Graz, Austria), and 0.1% Tween 80 as control treatment (7 groups, 16 specimens/group, naïve control group 34 specimens). Then, wireworms were singly kept in 200 ml

plastic cups filled with approximately 150g of the aforementioned, non-autoclaved field soil KL1. Five wheat seeds, soaked in sterile ddH_2O for one hour, were applied to each cup as a food source. Soil humidity was set to 20% gravimetrically by adding the required amount of sterile dH_2O via drenching. At least once a week, soil humidity was checked and adjusted to 20%, moldy seeds or seedlings were replaced, and soil-borne weed seedlings were removed. After four weeks under greenhouse conditions (25°C and 60% relative humidity), wireworms were checked for survival, and soil was searched for exuviae. Specimens that had pupated, died of bacterial infections (body soft and greenish), could not be found in the cup (suggested to have become imagos and escaped the cup), and imagos were removed from the analysis. Since the same soil was used for pretreatment and treatment, we assume effects of soil microbiota to be comparable between the test groups. Binomial testing was performed for each EPF strain separately to test whether mortality and the number of found cuticles differ due to a) immune priming (all three *Metarhizium* species combined), and b) species of the "treatment" EPF.

Methods Table M2: Test groups for the immune priming experiment in wireworms. Wireworms were exposed to *Metarhizium robertsii* in soil with low CFU concentration, then directly exposed to a spore solution (10^7 spores/ml) and kept in microcosms for four weeks. *: imagos, pupated, missing, and specimens with bacterioses at the end of the experiment were removed from analyses

Pre-treatment	Treatment	# valid specimen*
naïve	0.1% Tween (Control)	22
Pre-exposed (*M. robertsii)*	0.1% Tween (Control)	12
naïve	*M. anisopliae* strain A	11
naïve	*M. brunneum* strain B	8
naïve	*M. robertsii* strain R	11
Pre-exposed (*M. robertsii)*	*M. anisopliae* strain A	13
Pre-exposed (*M. robertsii)*	*M. brunneum* strain B	12
Pre-exposed (*M. robertsii)*	*M. robertsii* strain R	12

Additional References

1. Kabaluk T, Li-Leger E, Nam S (2017) Metarhizium brunneum – An enzootic wireworm disease and evidence for its suppression by bacterial symbionts. J Invertebr Pathol 150:82–87. https://doi.org/10.1016/j.jip.2017.09.012

2. Klausnitzer B (1994) 42. Familie: Elateridae. In: Die Larven der Käfer Mitteleuropas, Band L2, Myxophaga/Polyphaga, 1st ed. Springer Spektrum Berlin, Heidelberg, pp 118–189

3. Furlan L, Benvegnù I, Bilò MF, et al (2021) Species identification of wireworms (Agriotes

spp.; coleoptera: Elateridae) of agricultural importance in Europe: A new “horizontal identification table.” Insects 12:. https://doi.org/10.3390/insects12060534

4. Staudacher K, Pitterl P, Furlan L, et al (2011) PCR-based species identification of Agriotes larvae. Bull Entomol Res 101:201–210. https://doi.org/10.1017/s0007485310000337

5. Folmer O, Black M, Hoeh W, et al (1994) DNA primers for amplification of mitochondrial cytochrome c oxidase subunit I from diverse metazoan invertebrates. Mol Mar Biol Biotechnol 3:294–299

6. Rennstam Rubbmark O, Sint D, Horngacher N, Traugott M (2018) A broadly applicable COI primer pair and an efficient single-tube amplicon library preparation protocol for metabarcoding. Ecol Evol 8:12335–12350. https://doi.org/https://doi.org/10.1002/ece3.4520

7. Birer C, Tysklind N, Zinger L, Duplais C (2017) Comparative analysis of DNA extraction methods to study the body surface microbiota of insects: A case study with ant cuticular bacteria. Mol Ecol Resour 17:e34–e45. https://doi.org/10.1111/1755-0998.12688

8. Caporaso JG, Lauber CL, Walters WA, et al (2011) Global patterns of 16S rRNA diversity at a depth of millions of sequences per sample. Proc Natl Acad Sci 108:4516 LP – 4522

9. White TJ, Bruns T, Lee S, Taylor JW (1990) Amplification and direct sequencing of fungal ribosomal RNA genes for phylogenetics. In: Innis MA, Gelfand DH, Sninsky JJ, White TJ (eds) PCR Protocols: A Guide to Methods and Applications. San Diego: Academic Press, pp 315–322

10. Bolyen E, Rideout JR, Dillon MR, et al (2019) Reproducible, interactive, scalable and extensible microbiome data science using QIIME 2. Nat Biotechnol 37:852–857. https://doi.org/10.1038/s41587-019-0209-9

11. Martin M (2011) Cutadapt removes adapter sequences from high-throughput sequencing reads. EMPnet.journal 17:10. https://doi.org/doi:10.14806/ej.17.1.200.

12. Callahan BJ, McMurdie PJ, Rosen MJ, et al (2016) DADA2: High-resolution sample inference from Illumina amplicon data. Nat Methods 13:581

13. Rognes T, Flouri T, Nichols B, et al (2016) VSEARCH: A versatile open source tool for metagenomics. PeerJ 10:1–22. https://doi.org/10.7717/peerj.2584

14. Quast C, Pruesse E, Yilmaz P, et al (2013) The SILVA ribosomal RNA gene database project: Improved data processing and web-based tools. Nucleic Acids Res 41:590–596. https://doi.org/10.1093/nar/gks1219

15. Nilsson RH, Larsson KH, Taylor AFS, et al (2019) The UNITE database for molecular identification of fungi: Handling dark taxa and parallel taxonomic classifications. Nucleic Acids Res 47:D259–D264. https://doi.org/10.1093/nar/gky1022

16. McMurdie PJ, Holmes S (2013) Phyloseq: An R Package for Reproducible Interactive Analysis and Graphics of Microbiome Census Data. PLoS One 8:e61217. https://doi.org/10.1371/journal.pone.0061217

17. R Core Team (2018) R: A language and environment for statistical computing

18. Davis NM, Proctor DiM, Holmes SP, et al (2018) Simple statistical identification and removal of contaminant sequences in marker-gene and metagenomics data. Microbiome 6:1–15. https://doi.org/10.1186/s40168-018-0605-2

19. Kandlikar GS, Gold ZJ, Cowen MC, et al (2018) ranacapa: An R package and Shiny web app to explore environmental DNA data with exploratory statistics and interactive visualizations. F1000Research 7:1734. https://doi.org/10.12688/f1000research.16680.1

20. Oksanen J, Simpson GL, Blanchet FG, et al (2022) vegan: Community Ecology Package. R

package version 2.6-2

21. Martinez Arbizu P (2017) pairwiseAdonis: Pairwise multilevel comparison using adonis.

22. Segata N, Izard J, Waldron L, et al (2011) Metagenomic biomarker discovery and explanation. Genome Biol 12:R60. https://doi.org/doi:10.1186/gb-2011-12-6-r60

23. Guo K, Gao P (2021) microbial: Do 16s Data Analysis and Generate Figures

24. Knights D, Kuczynski J, Charlson ES, et al (2011) Bayesian community-wide culture-independent microbial source tracking. Nat Methods 8:761–765. https://doi.org/10.1038/nmeth.1650

25. Strasser H, Forer A, Schinner F (1996) Development of media for the selective isolation and maintenance of virulence of Beauveria brongniartii. Proc 3rd Int Work Microb Control Soil Dwell Pests 3rd Int Work Microb Control Soil Dwell Pests 125–130

26. Cernava T, Aschenbrenner IA, Grube M, et al (2015) A novel assay for the detection of bioactive volatiles evaluated by screening of lichen-associated bacteria. Front Microbiol 6:1–9. https://doi.org/10.3389/fmicb.2015.00398

Supplementary Figures

Supplementary Figure S1: Differences in bacterial (a-d) and fungal (e-h) diversity of soil, ecto- and endosymbionts. Species richness (**a,e**), Pielou's evenness (**b,f**), Shannon diversity (**c,g**) and PCoA plot of Bray Curtis dissimilarity (**d,h**) of soil (brown), ectosymbionts (orange) and endosymbionts (salmon). For Bray Curtis distances, PERMANOVA results using "habitat" as fixed effect to explain distances displayed on top.

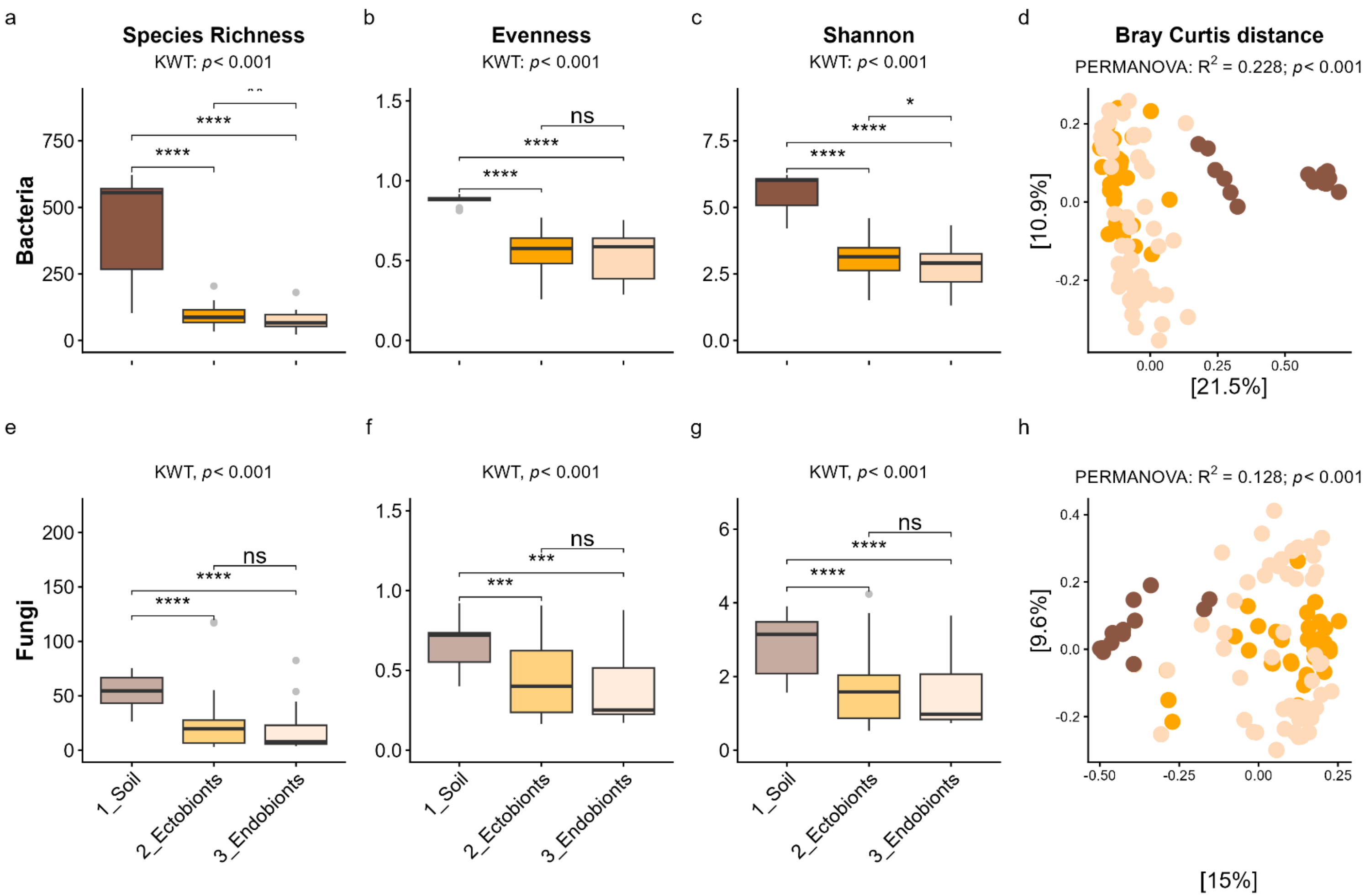

Supplementary Figure S2: Alpha diversity and microbial abundance as a function of wireworm size in ectosymbiotic communities. Linear models for bacterial (a-d; n = 35 and n = 12 for abundance) and fungal (e-f; n = 33 and n = 12 for abundance) species richness (a,e), Pielou's evenness (b,f), Shannon diversity (c,g) and abundance based on qPCR results (d,h) as a function of wireworm weight. Linear model with confidence interval, adjusted R^2 value with *p*-value < 0.05 displayed in corresponding graph.

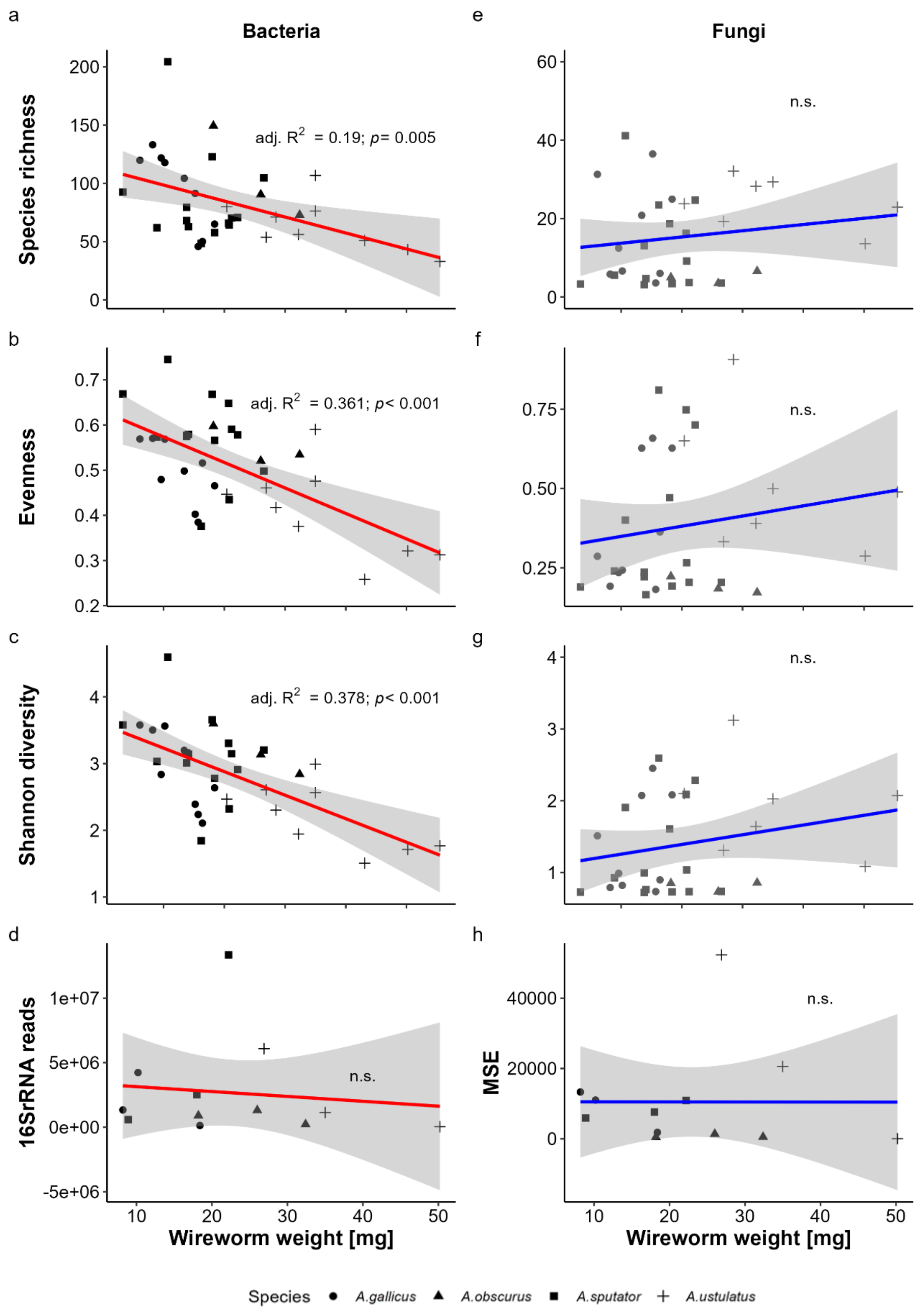

Supplementary Figure S3: Alpha diversity and microbial abundance as a function of wireworm size in endosymbiont communities. Linear models for bacterial (a-d; n = 35 and n = 12 for abundance) and fungal (e-f; n = 34 and n = 12 for abundance) species richness (a,e), Pielou's evenness (b,f), Shannon diversity (c,g) and abundance based on qPCR results (d,h) as a function of wireworm weight. Linear model with confidence interval, adjusted R^2 value with *p*-value < 0.05 displayed in corresponding graph.

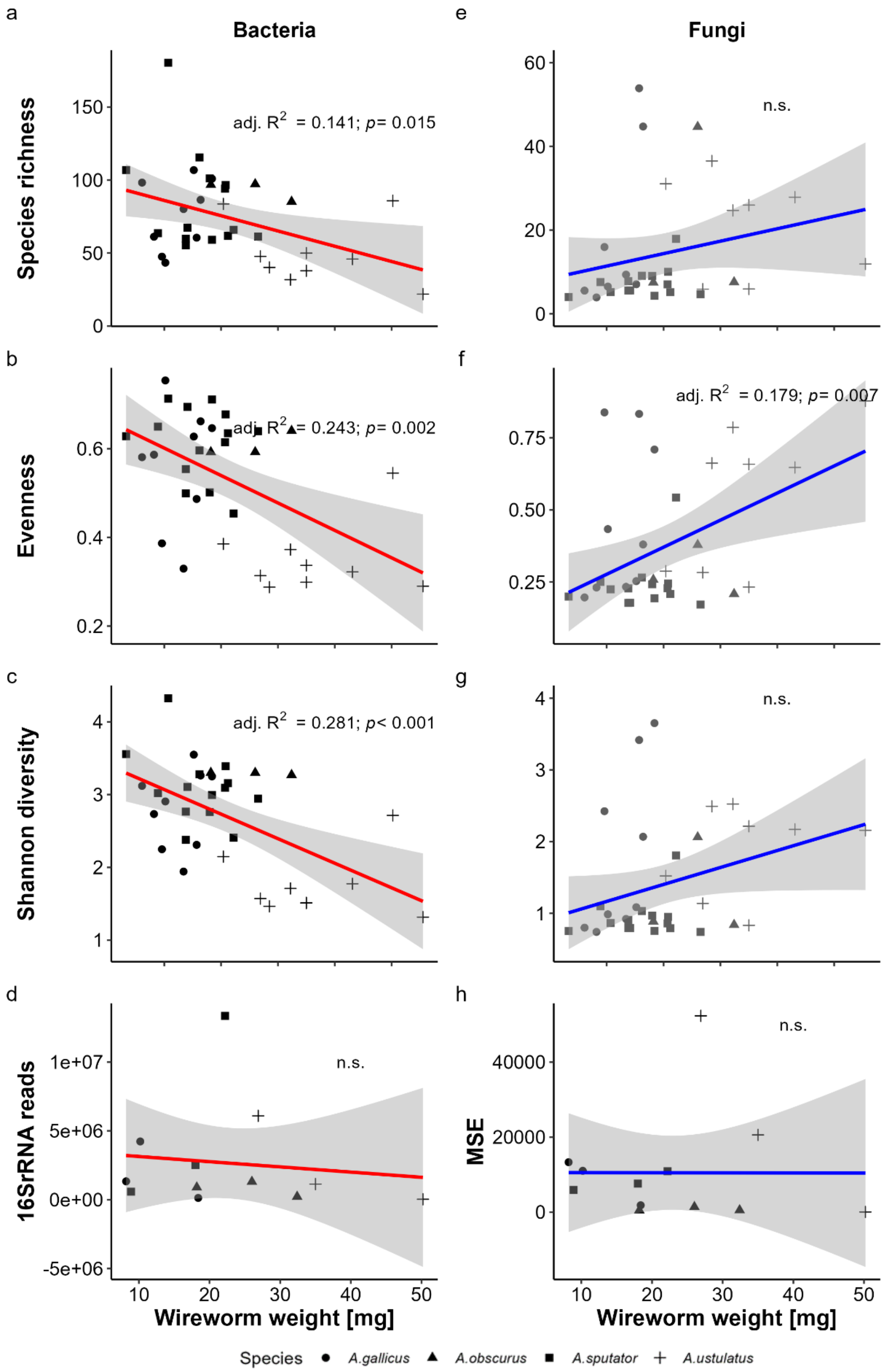

Supplementary Figure S4: Overview of alpha and beta diversity in wireworm endosymbionts. Species-dependent differences in bacterial (a-d) and fungal (e-h) species richness (a,e), Pielou's evenness (b,f), Shannon diversity (c,g) and Principal coordinate analysis plot of community composition based on Bray-Curtis dissimilarity (d,h). KWT: Kruskal Wallis test

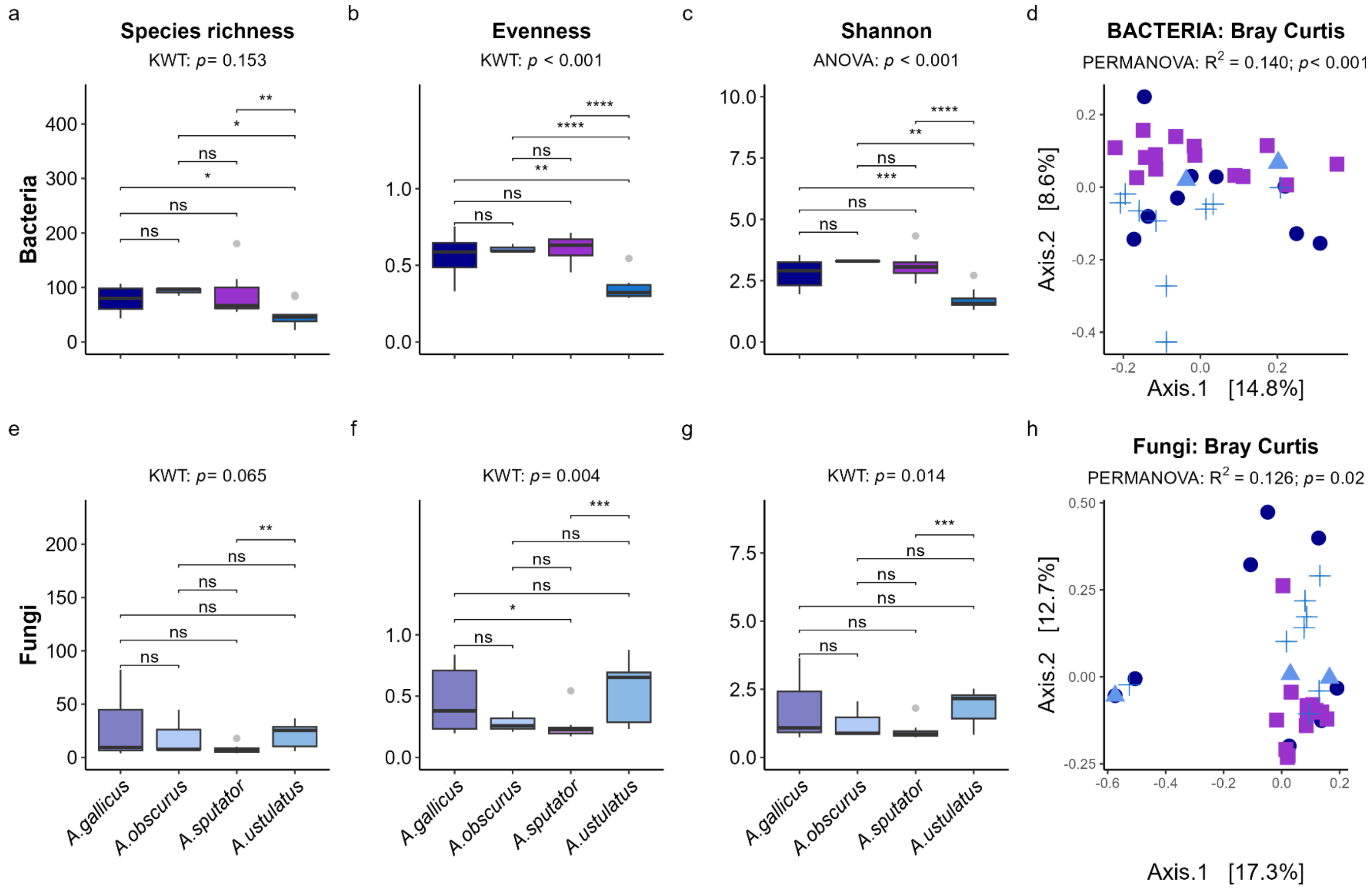

Supplementary Tables

Supplementary Table S1: Overview of filtered amplicon dataset

	**Total reads**	**Minimum reads**	**Maximum reads**	**Average reads**	**Average reads in soil**	**Average reads in ectosymbionts**	**Average reads in endosymbionts**
Bacteria	9,848,265	962	343,529	88,723	73,600	103,735	69,901
Fungi	7,503,947	5	358,132	68,844	4,948	61,800	109,725

Supplementary Table S2: Differences in soil diversity used in the current study. Pairwise comparison of species richness, Shannon diversity, Pielou's evenness (p-value for FDR-corrected Wilcoxon rank test, displayed as "value_bacteria/value_fungi), Bray-Curtis distances (bold: p-value < 0.05 according to pairwise PERMANOVA), and biomarker taxa with highest LDA according to linear discriminant analysis of effect size (LEfSe). Significant p-values highlighted in bold.

		**Soil 1 (KL2)**	**Soil 2 (KL3)**	**Soil 3 (CK)**	**Potting soil**
**Species richness** (KL2 > KL3 > CK >PS)	**Soil 1 (KL2)**	x			
	**Soil 2 (KL3)**	**0.043**/0.56	x		
	**Soil 3 (CK)**	**0.043**/0.29	0.24/0.56	x	
	**Potting soil**	**0.043**/0.091	0.34/0.091	**0.043**/**0.0024**	x
**Shannon diversity** (KL2 > KL3 > CK >PS)	**Soil 1**	x			
	**Soil 2**	0.24/0.83	x		
	**Soil 3**	0.057/0.76	0.086/0.83	x	
	**Potting soil**	0.057/0.12	0.057/0.13	0.34/**0.0019**	x
**Evenness** (KL3 > KL2 > CK > PS)	**Soil 1**	X			
	**Soil 2**	0.89/0.96	X		
	**Soil 3**	0.89/0.96	0.89/0.96	X	
	**Potting soil**	0.6/0.25	0.6/0.21	0.89/0.046	x
**Bray curtis**	**Soil 1**	X			
	**Soil 2**	**R^2_{bac} = 0.239;** R^2_{fun} = 0.164	X		
	**Soil 3**	**R^2_{bac} = 0.226;** R^2_{fun} = 0.235	**R^2_{bac} = 0.227; R^2_{fun} = 0.483**	X	
	**Potting soil**	**R^2_{bac} = 0.476;** R^2_{fun} = 0.248	**R^2_{bac} = 0.475;** R^2_{fun} = 0.540	R^2_{bac} = 0.268**;** **R^2_{fun} = 0.704**	x
**BIOMARKER**		Ca. *Udaeobacter*	*Vicinamibacterales* indet.	*Bradyrhizobium*	*Chitinophagaceae* indet.
		Gaiellales indet.	*Bacillus*	*Sphingomonas*	*Glutamicibacter*
		Gaiella;	*Xiphinematobacter;*	*Gemmatimonas;*	*Asanoa;*
		Nectriaceae indet.	*Solicoccozyma*	*Hypocreales* indet.	*Meliniomyces*
		Sordariomycetes indet.		*Mortierella*	

Supplementary Table S3: Pairwise comparisons of bacterial and fungal community composition (pairwise PERMANOVA of Bray-Curtis distances) of the three tested compartments (soil, ectobionts, endobionts) **.**

	Compartment 1	Compartment 2	SumOfSquares	R^2	F	Pr(>F)
**Bacteria**	soil (n = 16)	ectobionts (n = 60)	4.4262	0.22513	21.5	<0.001
	soil (n = 16)	endobionts (n = 35)	4.4268	0.32731	23.842	<0.001
	ectobionts (n = 60)	endobionts (n = 35)	1.1927	0.06634	6.608	<0.001
**Fungi**	soil (n = 16)	ectobionts (n = 60)	2.8878	0.13428	10.237	<0.001
	soil (n = 16)	endobionts (n = 35)	2.9677	0.21312	12.459	<0.001
	ectobionts (n = 60)	endobionts (n = 35)	0.7377	0.02924	2.5901	<0.001

Supplementary Table S4: Summary of RT-qPCR results for 16SrRNA and ITS reads in wireworms. Log_{10}-transformed reads and spore equivalents using primers for 16SrRNA and ITS for bacteria and fungi, respectively. MSE: *Metarhizium* spore equivalents

		minimum	mean	maximum
**Bacteria**	**Ectosymbionts**	4.34	6.18	6.75
	**Endosymbionts**	4.55	6.42	7.13
**Fungi (MSE)**	**Ectosymbionts**	0.78	3.83	4.02
	**Endosymbionts**	1.52	4.02	4.72

Supplementary Table S5: ASVs shared between ectosymbiotic and endosymbiotic communities based on VENN analyses. Number of unique ASVs, percent of unique ASVs to all ASVs in brackets, and mean relative abundance of ectosymbiotic or endosymbiotic communities, separated by wireworm species. Low numbers of unique ASVs in *A. obscurus* may arise from the lower sample size compared to the other tested species.

		**Ectosymbionts**		**Endosymbionts**	
		# unique ASV (%)	Mean rel. abundance [%]	# unique ASV (%)	Mean rel. abundance [%]
**Bacteria in**	*A.*cf. *gallicus*	1481 (67.7%)	2.2	597 (45.8%)	1.3
	A. obscurus	720 (77.3%)	6.7	202 (94.4%)	1.5
	A. sputator	1449 (71.7%)	1.0	895 (61.1%)	1.7
	A. ustulatus	1516 (82.1%)	2.8	663 (66.7%)	1.0
**Fungi in**	*A.*cf. *gallicus*	368 (55.1%)	3.6	661 (68.8%)	14.5
	A. obscurus	125 (54.1%)	0.2	604 (66.4%)	5.7
	A. sputator	397 (72.1%)	4.6	243 (61.2%)	1.1
	A. ustulatus	556 (69.7%)	21.8	398 (62.2%)	15.1

Supplementary Table S6: Prevalence and abundance of the genus Metarhizium in four field-collected wireworm species. The genus *Metarhizium* was represented by *M. anisopliae* and *M. marquandii* (synonym of *Marquandomyces marquandii*). *M. anisopliae* was more abundant than *M. marquandii* (all samples merged) by factors of 494 and 53 in ectosymbiotic and endosymbiotic samples, respectively.

		A.* cf. *gallicus	***A. obscurus***	***A. sputator***	***A. ustulatus***
**Ectosymbionts**	**Prevalence**	5 of 9	2 of 3	8 of 14	20 of 32
	**Rel. Abundance [%]**	0.64	0.01	0.92	1.3
**Endosymbionts**	**Prevalence**	4 of 9	2 of 3	10 of 14	6 of 9
	**Rel. Abundance [%]**	0.87	0.2	0.013	1.07

Supplementary Table S7: Top five potential biomarkers for wireworm species in wireworm cuticles according to LefSe analyses. see separate file

Supplementary Table S8: Top five potential biomarkers for wireworm species in wireworm whole body according to LefSe analyses: see separate file

Supplementary Table S9: Potential legacy effect of origin soil on *Agriotes ustulatus* microbiome diversity after soil swap. Tested model and corresponding p-value in ectosymbionts and endosymbionts. Significant values highlighted in bold. T7: sampling timepoint after soil swap (day 213) after 19 days in potting soil; KWT: Kruskal-Wallis test; PERMANOVA: permutational analysis of variance.

	**Model**	**Ectosymbionts (T7) p-value**	**Endosymbionts (T7) p-value**
**Bacteria**	Species richness ~ origin soil	ANOVA: **p = 0.031**	ANOVA: p = 0.393
	Evenness ~ origin soil	ANOVA: p = 0.294	ANOVA: p = 0.670
	Shannon ~ origin soil	ANOVA: p = 0.303	ANOVA: p = 0.872
	Bray Curtis distance ~ origin soil	PERMANOVA: p = **0.026**	PERMANOVA:p = 0.118
**Fungi**	Species richness ~ origin soil	KWT: p = 0.351	ANOVA p = 0.082
	Evenness ~ origin soil	ANOVA: p = 0.310	ANOVA p = 0.987
	Shannon ~ origin soil	ANOVA: p = 0.787	ANOVA p = 0.812
	Bray Curtis distance ~ origin soil	PERMANOVA: p = 0.751	PERMANOVA: p = 0.5477

Supplementary Table 10: Test groups and raw data for the experiment regarding immune priming in wireworms. Effect of low concentrations of EPF spore exposure (soil drenching) followed by high concentrations (exposure in 10^7 spores/ml) treatment on wireworm mortality and number of cuticles found in soil. *: imagos, pupated, missing, and specimens with bacterioses at the end of the experiment were removed from analyses; mycoses: wireworms that were dead and visually infected with *Metarhizium*.

**Pre-treatment**	**Treatment**	**# valid specimen***	**mycoses [%]**	**# cuticles in soil**
naïve	0.1% Tween (Control)	22	0	1
Pre-exposed (*M. robertsii)*	0.1% Tween (Control)	12	25	1
naïve	*M. anisopliae* strain A	11	27	8
naïve	*M. brunneum* strain B	8	25	9
naïve	*M. robertsii* strain R	11	27	3
Pre-exposed (*M. robertsii)*	*M. anisopliae* strain A	13	8	3
Pre-exposed (*M. robertsii)*	*M. brunneum* strain B	12	8	6
Pre-exposed (*M. robertsii)*	*M. robertsii* strain R	12	25	8

*: imagos, pupated, missing, and specimens with bacterioses at the end of the experiment were removed from analyses
